# SWnet: a deep learning model for drug response prediction from cancer genomic signatures and compound chemical structures

**DOI:** 10.1186/s12859-021-04352-9

**Published:** 2021-09-10

**Authors:** Zhaorui Zuo, Penglei Wang, Xiaowei Chen, Li Tian, Hui Ge, Dahong Qian

**Affiliations:** 1 Institute of Medical Robotics, Shanghai Jiao Tong University, 2F of the Translational Medicine Building, No. 800 Dongchuan Road, Shanghai, 200000 China; 2 Novartis Institutes for Biomedical Research, 4218 Jinke Road, Pudong, Shanghai, 201203 China

**Keywords:** Deep learning, Genomics, Cheminformatics, Drug response

## Abstract

**Background:**

One of the major challenges in precision medicine is accurate prediction of individual patient’s response to drugs. A great number of computational methods have been developed to predict compounds activity using genomic profiles or chemical structures, but more exploration is yet to be done to combine genetic mutation, gene expression, and cheminformatics in one machine learning model.

**Results:**

We presented here a novel deep-learning model that integrates gene expression, genetic mutation, and chemical structure of compounds in a multi-task convolutional architecture. We applied our model to the Genomics of Drug Sensitivity in Cancer (GDSC) and Cancer Cell Line Encyclopedia (CCLE) datasets. We selected relevant cancer-related genes based on oncology genetics database and L1000 landmark genes, and used their expression and mutations as genomic features in model training. We obtain the cheminformatics features for compounds from PubChem or ChEMBL. Our finding is that combining gene expression, genetic mutation, and cheminformatics features greatly enhances the predictive performance.

**Conclusion:**

We implemented an extended Graph Neural Network for molecular graphs and Convolutional Neural Network for gene features. With the employment of multi-tasking and self-attention functions to monitor the similarity between compounds, our model outperforms recently published methods using the same training and testing datasets.

**Supplementary Information:**

The online version contains supplementary material available at 10.1186/s12859-021-04352-9.

## Background

In the past decade, the concept of personalized medicine has been widely accepted with the advance of molecular medicine and genomics, seeking to underpin the association between individuals’ biological background and drugs response. Identification of molecular biomarkers is now a common practice in clinical studies, especially in the field of cancer therapy. The availability of genomics data and therapeutic agents make cancer an ideal field for the study of precision medicine that aims to match patient’s molecular background with the selection of drugs. In addition, understanding the interplay between biology and drugs chemical properties is also a key to the practice of drug repositioning. Research in the field of cancer has gained deep insights into its molecular features [1–3]. While clinical studies of drug response is expensive, a comprehensive catalogue of stable cancer cell lines that captures patient’s molecular features has been used for large-scale in vitro screening of anticancer drugs [4–6]. Data from these studies provide rich sources for investigating the nature of drug–gene interaction, and can provide meaningful guidance to clinical practice.

Since large-scale screening datasets have become publicly available, the computational community has made great efforts in developing predictive models that link gene-level signatures directly with drug response [7]. A variety of machine learning techniques [8] have been evaluated, including elastic net, regularized matrix factorization, sparse-linear/non-linear regressions, kernel methods, network-based methods, and ensemble models. Valuable insights were gained from these studies, such as the predictive power of different genomic data types, the performance of different algorithms and the benefit of incorporating prior knowledge [9–14]. Chemical structures can also be used to predict drug activity in a comparable fashion [15]. Some studies have tried to integrate genomic and chemical signatures in a predictive model. Several previously published methods showed that combining chemical structural information and cell lines’ molecular profile could improve $IC_{50}$ prediction accuracy [16–22].

We have developed a novel deep-learning predictive model, self-attention gene weight layer network (SWnet), that aims to leverage the current advancement of machine learning methods and integrate genomics and cheminformatics. Our method has unique characteristics compared to existing methods: (1) with the application of Graph Neural Network (GNN), we converted the 2D representations of chemicals into continuous vectors in the latent space; (2) we used the gene weight layer to combine the information of gene mutation and gene expression, and then used the multi-task model to extract the interaction information of chemical structure and genetics to improve the prediction accuracy; (3) we applied self-attention mechanism [23] to incorporate the structural similarity between compounds into model training.

SWnet was trained and validated using the Genomics of Drug Sensitivity in Cancer (GDSC) dataset [5] and Cancer Cell Line Encyclopedia (CCLE) dataset [24]. We compared our result to a few recent studies that represent the most advanced methods in the field and showed that our predictive model outperformed other methods trained on the same dataset.

## Methods

### Training and testing data

Genomics of Drug Sensitivity in Cancer (GDSC) is the largest public resource of anticancer drug sensitivity screening using over 1000 human cancer cell lines. Drugs in GDSC are comprised of approved drugs, experimental drugs in clinical trials, and tool compounds. The Cancer Cell Line Encyclopedia (CCLE) is another commonly used resource with genomic profiling of cancer cell lines, which allows predictive modelling of anticancer drug sensitivity [1, 24]. The structures of compounds in GDSC and CCLE were obtained from PubChem or ChEMBL [25, 26]. The Simplified Molecular Input Line Entry System (SMILES) was converted to Morgan fingerprints using RDKit [27] and the similarity between compounds was determined by Tanimoto distance. The structures of compounds from GDSC belong to 209 Bemis-Murcko scaffold and can be further grouped into 56 clusters based on chemical fingerprints (with linkage type of UPGMA, and distance of 0.6 by Tanimoto distance), showing a high level of chemical diversity.

Sensitivity is measured by the natural log-transformed half maximal inhibitory concentration ($IC_{50}$). Cell lines have been extensively characterized at molecular level. We used gene expression and genetic mutation as biological features in our model training. The original expression matrix (RMA value from Array Express) was normalized to z-score per cell line. The binary mutation matrix was produced by collapsing all the somatic nonsynonymous mutations for each gene, regardless of the genomic location.

The high dimension of the genomic feature matrices is likely to cause overfitting. Subsets of genes based on biological functions could be selected to reduce the dimension, as proposed by other studies [12, 28]. We selected genes based on the following criteria: (1) relevance to cancer based on the Catalogue of Somatic Mutations in Cancer (COSMIC) database [3]; (2) gene expression showing non-redundancy, which means that a subset of genes are selected to represent the whole expression profile of the transcriptome. According to Broad L1000 project [6], this set of genes has been shown to be sufficient to predict the transcriptome change upon drug treatment. We used the gene set to reduce the possibility of overfitting. Finally, we merged the two gene lists and obtained 1478 genes across 1018 cell lines, whose expression and mutation represent the genomic features in model training.

### Model architecture

#### Dual convergence model architecture

**Fig. 1:**
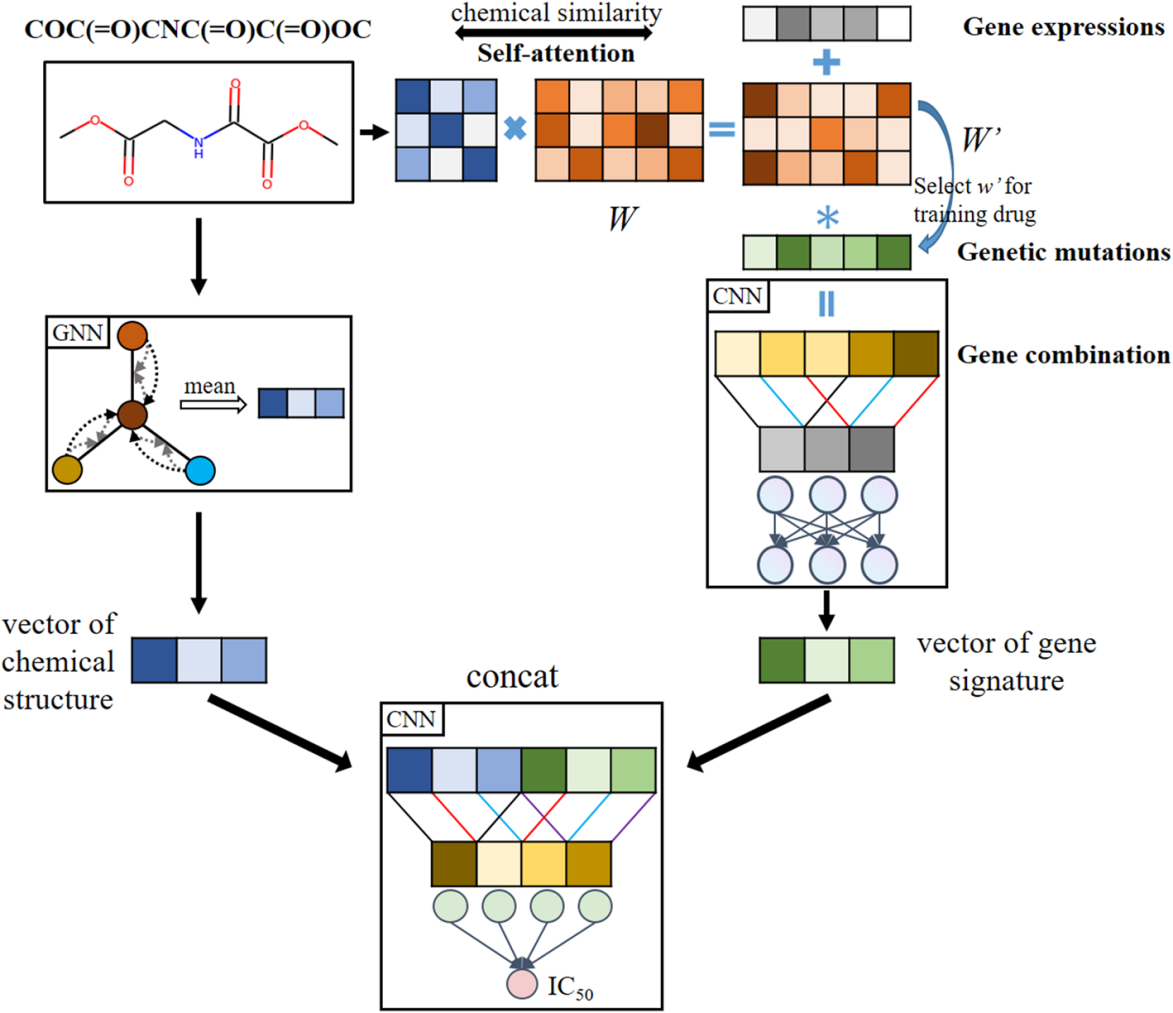
The overview of model architecture. Chemical structure, gene expression and genetic mutation serve as input of the model. Graph neural network encoded the drugs and convolutional neural network extracted gene expression and genetic mutation features simultaneously. Through self-attention, we incorporated the chemical similarity into the input to train gene weight layer $W'$. Gene weight layer $W'$ combined gene expression, genetic mutation and drug similarity. Then the model concatenated drug vector and genetic vector to predict the $IC_{50}$

Our deep-learning model adopted a dual convergence architecture as shown in Fig. 1, which means the mutations and expression of genes and chemical structures of drugs information were modelled separately since they contained different types of information. The two were later merged into one prediction model. Genomic signatures and molecular graphs were processed in parallel through GNN and Convolutional Neural Network (CNN) layers to extract independent features, which were then concatenated. The gene branch in SWnet used 3 convolutional layers and 1 fully connected layer to generate gene embeddings. Then the embeddings of molecule and gene were concatenated and input into a prediction subnetwork, which consists of convolutional layer, pooling layer and batch normalization layer, to extract high-level features. Finally, the high-level features were input to fully connected layer to predict $IC_{50}$. It is noted that we used 1D CNNs in gene branch and prediction subnetwork to decrease the number of trainable parameters, therefore reducing the complexity of our model to avoid the risk of overfitting and improve the model performance. Based on dual convergence architecture, we also integrated multi-task learning and self-attention mechanism to further improve the performance. We described the architecture in more details in the following sections.

#### Parsing of compound chemical structures using graph method

There are many representations of chemical structures in the field of machine learning, such as molecular fingerprints, text-based representations (SMILES/INCHI), graph-based and 3D structure & surface. There are two main methods to encode molecules using continuous embedding vectors. One method is to use RNNs in variants autoencoder deep-learning network to generate continuous embedding vectors from SMILES [29, 30]. The other method is to learn features from graph-structured inputs [31–33].

In our study, we implemented an extended Graph Neural Network (GNN) [34] for molecular graphs. Normally, we represent a graph as $G = (V,E)$, where *V* is the set of nodes and *E* is the set of edges. For a molecule, $v_i \in V$ represents the *i*th atom and $e_{ij} \in E$ represents the chemical bond between *i*th atom and *j*th atom. The GNN takes a graph *G* as input, then produces a graph-level representation $h_G \in R^d$. In our extended GNN, we used *r*-radius subgraphs [9] to solve the issue that representation learning was ineffective due to the low model complexity. The *r*-radius subgraphs were induced by the neighboring nodes and edges within radius *r* from a node. Different from that in normal GNNs, in the extended GNN, we randomly initialized embedding for *r*-radius node and *r*-radius edge, and then updated the representation by backpropagation.

**Fig. 2:**
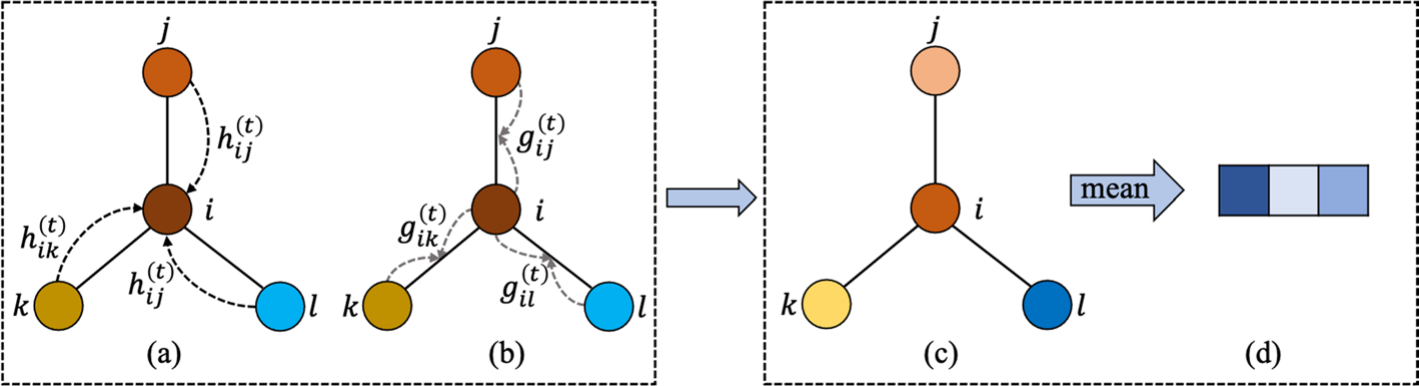
The overview of GNN architecture. **a** The process of updating node embedding through neighboring nodes. **b** The process of updating embedding through side node embedding. **c** The final graph after the transition function. **d** The graph-level representation through arithmetic mean of all node embeddings

The process of GNN for molecular graphs can be described as a transition function and an output function. Figure 2 illustrates the general GNN architecture for molecular graphs. The transition function iteratively updates the node information by combining its neighboring nodes and edges, and the output function maps the node-level feature vectors to graph-level representation. The extended GNN has transition function for nodes and edges respectively, and updates the current node embedding through leveraging previous node and edge embeddings. For edge transition, we updated the edge embedding $e_{ij}^{(t+1)}$ through both end node embeddings $v_i^{t+1}$ and $v_j^{t+1}$ as follows:

1 $$\begin{aligned} e_{ij}^{(t+1)}&= \sigma (e_{ij}^{(t)}+g_{ij}^{(t)}) \end{aligned}$$

2 $$\begin{aligned} g_{ij}^{(t)}&= f(w_e(v_i^{(t)}+v_j^{(t)}+b_e)) \end{aligned}$$

where $\sigma$ is the element-wise sigmoid function and *f* is a non-linear activation function like ReLU, $w_e \in R^{d \times d}$ and $b_e \in R^d$ are the trainable parameters and bias vector respectively, *d* is the dimension of edge embedding vector.

We used similar iterative procedure for node transition, and we update the *i*th node embedding $v_i^{t+1} \in R^d$ at time step $t+1$ through the following transition function:

3 $$\begin{aligned} v_i^{(t+1)}&= \sigma \left( v_i^{(t)}+\sum _{j \in N(i)}h_{ij}^{(t)}\right) \end{aligned}$$

4 $$\begin{aligned} h_{ij}^{(t)}&= f\left( w_n \begin{bmatrix} v_j^{(t)}\\ e_{ij}^{(t)} \end{bmatrix} +b_n\right) \end{aligned}$$

where $w_n \in R^{d \times 2d}$ and $b_n \in R^d$ are the trainable parameter matrix and bias vector respectively, and *N*(*i*) is the set of neighboring indices of *i*. The extended GNN computes the *i*th node embedding through combining the edge embedding and the neighboring node embedding iteratively, and the node gradually obtains global information on the graph.

The next operation takes the graph-level representation as input which is from the output function. Our output function is an arithmetic mean of all node embedding from transition function as follows:

5 $$\begin{aligned} h_{G}=\frac{1}{N}\sum _{i=1}^{N}v_i^{(t)} \end{aligned}$$

where *N* is the number of nodes in the graph. While this is a simple operation to obtain the graph-level representation, it works well in practice.

The graph-level representation extracted by GNN embedded the 2D structure information of molecules into the feature vector, compared with the binary fingerprints used by other methods [15, 35], it effectively avoids the problem of sparse features. We think the graph-level representation extracted by GNN can benefit our task.

#### Weight vector/matrix to evaluate the significance of genetic mutation

**Fig. 3:**
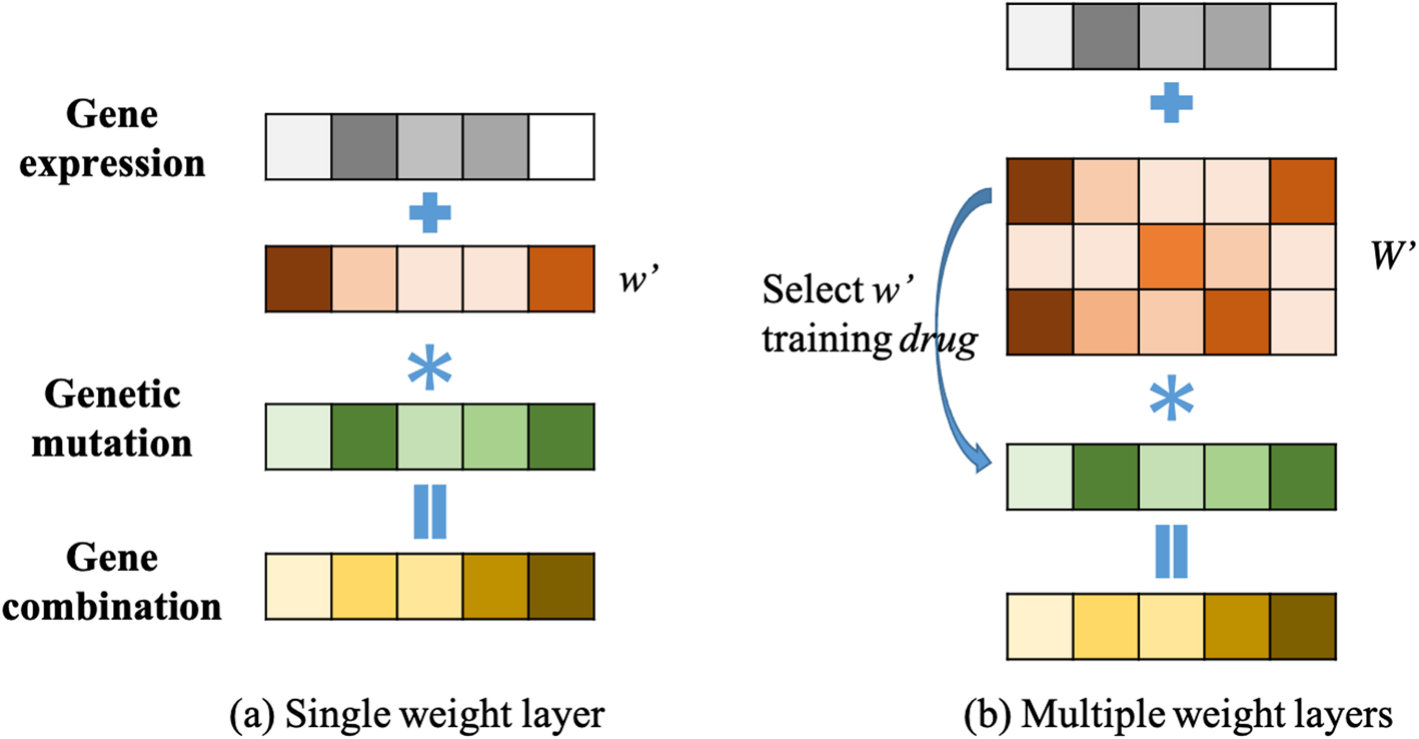
Gene weight layer. Gene feature was combined as the sum of normalized gene expression value and a weight adjusted genetic mutation value. Two ways of generating the mutation component were tested. **a** Single weight layer: a common weight vector $w'$ was applied to all genetic mutations without considering the heterogeneity of gene-drug relationship. **b** Multiple weight layers: a weight matrix $W'$ was applied to account for the heterogeneity of gene-drug relationship so that each drug would have its own weight vector for genetic mutation

Most published research use only gene expression data or only genetic mutation data to train models [18]. We wanted to integrate these two data types, so we added a weight layer to the deep learning model. In this way, the mutation status of each gene is fed into the model with a weight that can be trained. As shown in Fig. 3, a combined feature for gene is calculated as:

6 $$\begin{aligned} geneCom = geneExp + geneVar * {w'} \end{aligned}$$

where *geneCom* is the combination of expression (*geneExp*) and mutation (*geneVar*). $w'$ represents the weight for genetic mutation. $*$ represents the vector dot product. The feature of each gene is the gene expression value plus the product of genetic mutation value and weight by Eq.(6). We have two kinds of weights: one is single weight vector whose dimension is *w* (1, 1478), and the other is multi-weight vector whose dimension is *W* (*N*, 1478). *N* is the number of anticancer drugs. Thus, for each compound, we trained for a vector *w* (1, 1478) that represented the significance of mutant genes. In the later section, we will show how to train the multi-layer weight matrix.

#### Multi-task learning

Multi-task learning (MTL) problem is defined as multiple supervised learning tasks considered together [12]. In the previous section, we described a multi-layer weight matrix to measure the interaction between individual compound and genetic mutation. Each layer (vector of size $1*1478$) is thus independent, and the simplest approach would be to solve these tasks independently through separating the dataset by compounds. However, it has been shown in previous studies that MTL can improve the performance [12, 36]. Since each task has the same model structure, we trained the 221 tasks all at once, using a common MSE loss for all drugs.

#### Self-attention

For the multi-task model described in previous section, each drug has its own gene weight parameters, which are supposed to be relevant to the drug itself. But we believe that similar drugs may have similar gene weight parameters. To take into consideration the similarity between drugs’ chemical structures, we applied a self-attention method, which uses drug similarity matrix to update the parameters of gene weight layer.

An attention function can be described as a query and a set of key-value pairs to an outputs, where the query, keys, values and output are all vectors. The output is computed as a weighted sum of the values, where the weight assigned to each value is computed by compatibility of the query with the corresponding key [23].

7 $$\begin{aligned} Attention(Q,K,V)=softmax(a(Q,K))V \end{aligned}$$

*Q*, *K* represent SMILES of compounds. *V* represents weight parameters of gene weight layer. *a*(*Q*, *K*) is an alignment function [20] which gives scores how well the inputs and the outputs match, and we normalized the scores by softmax function. The softmax function is a normalized exponential function as follows:

8 $$\begin{aligned} softmax(x)_i = \frac{e^{x_i}}{\sum _{j=1}^{d}e^{x_j}}, \quad for \; i=1,\ldots ,d \; and \; x=(x_1,\ldots ,x_d) \in R^d \end{aligned}$$

**Fig. 4:**
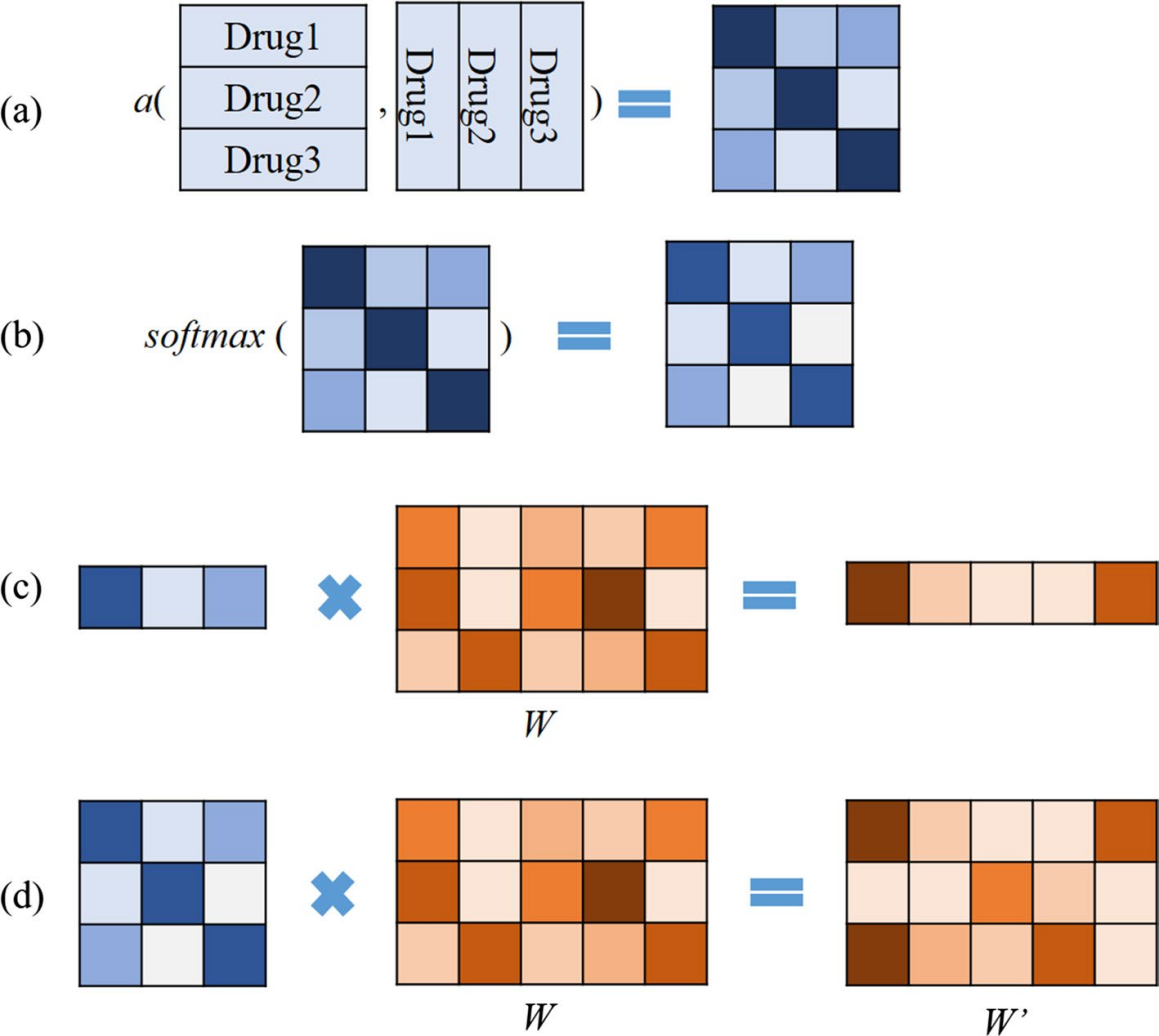
Self-attention calculation process. **a** Function *a* calculates the similarity between drugs. **b** The softmax function calculates the proportion of similarity score. **c** For one compound, through matrix multiplication, the weight layer will have compound similarity features. **d** The $W'$ layer will be updated during the training process and finally reflects the contribution of genetic mutation to each drug’s sensitivity. By multiplying the *W* layer with the softmax matrix, drug–drug similarity is taken into account

In our model, the calculation process is shown in Fig. 4. First, we used RDKit Tools to calculate the similarity of Morgan fingerprints between compounds to obtain *a*(*Q*, *K*). Then we performed matrix multiplication with the weight matrix. *a*(*Q*, *K*) represents the similarity matrix between all compounds. The output $W'$ has the same dimension as *W*, but contains drug similarity information. Figure 1 illustrates the self-attention mechanism for $IC_{50}$ prediction.

For the drug response prediction of a new drug, its corresponding *w* could be calculated from the trained $w'$. The calculation process is described in Fig. 4a–c. This way, the drug response prediction for new drugs will be more accurate.

### Other methods for comparison

We selected four previously published methods for comparison. We used Mean Square Error (MSE) and $R^2$ to evaluate the performance of models. The MSE metric measures the average squared difference between the predicted values $\hat{y_i}$ and the actual values $y_i$, it is calculated as follows:

9 $$\begin{aligned} MSE = \frac{1}{N}\sum _{i=1}^{N}(y_i-\hat{y_i})^2 \end{aligned}$$

where the *N* represents the number of samples. The $R^2$ quantifies the degree of any linear correlation between the predicted values $\hat{y_i}$ and the actual values $y_i$, it is calculated as follows:

10 $$\begin{aligned} R^2 = 1-\frac{\sum _{i}^{N}(y_i-\hat{y_i})^2}{\sum _{i}^{N}(y_i-{\bar{y}})^2}, \quad where \; {\bar{y}} = \frac{1}{N}\sum _{i}^{N}y_i \end{aligned}$$

These methods were all trained and tested on GDSC and CCLE datasets.

1. Kernelized Bayesian multi-task learning (KBMTL). This method formulated a novel Bayesian algorithm that combines kernel-based non-linear dimensionality reduction and binary classification or regression [11]. The joint Bayesian formulation projects data points into a shared subspace and learns predictive models for all drugs in this subspace. In our paper, we used the Gaussian kernel with $\sqrt{25000}$ kernel width to calculate similarity between cell lines. The parameters were ($\alpha _\lambda$, $\beta _\lambda$, $\alpha _\epsilon$, $\sigma _h$, $\sigma _h$, R) = (1,1,1,1,0.1,1,10) and we used 200 iterations in all the experiments for KBMTL.
2. Similarity-regularized matrix factorization (SRMF). This method predicted anticancer drug responses of cell lines by using chemical structures of drugs and baseline gene expression levels in cell lines [14]. Specifically, chemical structural similarity of drugs and gene expression profile similarity of cell lines were considered as regularization terms, which were incorporated to the drug response matrix factorization model. We used Morgan fingerprint to calculate the drug similarity matrix in our paper. And the regularization parameters in SRMF were (*K*, $\lambda _l$, $\lambda _d$, $\lambda _c$) = (45, $2^{-3}$, 0, $2^{-3}$). Finally, we used 50 iterations in all the experiments for SRMF.
3. Weighted Graph Regularized Matrix Factorization (WGRMF). This method constructed a *p*-nearest neighbor graph to sparsify drug similarity matrix and cell line similarity matrix, respectively [37]. Using the sparse matrices in the graph regularization terms, it performed matrix factorization to generate the latent matrices for drugs and cell lines in anticancer drug response prediction task. We used Morgan fingerprint to calculate the drug similarity matrix in our paper. The regularization parameters used in our paper were (*k*, *p*, $\lambda _l$, $\lambda _d$, $\lambda _c$) = (182, 20, 1, 2, $2^{-5}$) for GDSC dataset and (*k*, *p* $\lambda _l$, $\lambda _d$, $\lambda _c$) = (24, 10, 1, 2, 1) for CCLE dataset. Finally, we used 50 iterations in all the experiments for WGRMF.
4. Cancer Drug Response profile scan (CDRscan). This paper employed a deep learning dual convergence architecture, where the genomic mutational fingerprints of cell lines and the molecular fingerprints of drugs were processed individually, and then merged by ‘virtual docking’, an in silico modelling of drug treatment [35]. By inputting molecular fingerprints of drugs, they achieved better $IC_{50}$ prediction result.
5. Graph convolutional network for drug response prediction (GraphDRP). In GraphDRP [22], drugs were represented in molecular graphs directly capturing the bonds among atoms. Meanwhile, cell lines were depicted as binary vectors of genomic aberrations. The features of drugs and cell lines were learned by the convolutional layers, and then the combination of drug and cell line features were used to predict $IC_{50}$ value. In our paper, we used GIN graph model in GrapDRP to learn the representation of the drug in GDSC and CCLE datasets.

## Results

In this section, we showed the performance of our method on GDSC and CCLE datasets. Meanwhile, we studied the effect of gene weight layer, multi-task, self-attention and different *r*-radius GNN for our model on GDSC dataset. We designed three experiments. The first is the initial model with single matrix of gene weight layer. The second is MTL model with multiple matrices of gene weight layer. The third is self-attention model base on MTL but with increased similarity feature of drugs. The three experiments are with different *r*-radius (*r* = 1, 2, 3). As shown in Table 3, after introducing the self-attention mechanism, the performance has been significantly improved. In addition, the trained gene weight layer helped to identify genes whose mutation status may strongly influence the prediction of drug ecacy.

### Performance on GDSC and CCLE datasets

GDSC data was pre-processed according to Methods Section Training and testing data. The final GDSC dataset consists of 983 cell lines with 1478 gene-level signatures and 221 drugs. A total of 177,128 instances of “chemical structure + gene signature vs. drug sensitivity” were in the final input for training the deep learning models (removing missing values where drug sensitivity is not available). The CCLE dataset consists of 24 compounds, covering 469 distinct cell lines for a total of 10853 instances.

In order to train and test the model, we randomly divided the GDSC dataset into the training (159415 instances) and testing (17,713 instances) sets, and split the CCLE dataset into the training (9767 instances) and testing (1086 instances) sets, which corresponded to 90% and 10% of the total instances, respectively.

Compared with previously published methods, our deep learning model shows superior predictive performance (Table 1). Other models extracted features in all gene expression to achieve good predictive performance, but it is very difficult to extract really effective features from so many gene expression datasets. Different from other papers, CDRscan used mutation position to train model. They selected sequence variation information at 28328 positions from 567 genes in CGC [38].

**Table 1:** Comparison of performances with previously published methods

Dataset	Model	MSE	$R^2$
GDSC	KBMTL	1.2642	0.8225
	SRMF	0.9874	0.8614
	WGRMF	0.9844	0.8618
	GraphDRP	1.2586	0.8229
	CDRscan	2.1525	0.6978
	SWnet	**0.9384**	**0.8683**
CCLE	KBMTL	1.9480	0.5439
	SRMF	1.2565	0.7058
	WGRMF	1.3026	0.6950
	GraphDRP	1.3121	0.6928
	CDRscan	1.1960	0.7200
	SWnet	**1.1604**	**0.7283**

Bold values represents the result is the best performance among the models participating in the comparison

### Single weight layer

In this section, we discussed the effectiveness of genetic mutation features and gene weight layer. To study the influence of genetic mutation on model performance, we trained SWnet without gene weight layer. In Table 2, we input two data types into SWnet gene branch: (1) gene expression only; (2) gene expression plus genetic mutation. As shown in Table 2, the best MSE is 0.9727 when the gene expression features as input and $r = 2$ for radius. Meanwhile, the best MSE is 0.9853 when the gene expression and genetic mutation as input and $r = 2$ for radius. From the results we could see that the introduction of genetic mutation through simple addition would change the distribution of gene expression data and lead to the deterioration of training results.

**Table 2:** Performance of model training without gene weight layer in different *r*-radius

Features	*r*-radius	MSE	$R^2$
Gene expression	1	1.0765	0.8489
	2	**0.9727**	**0.8634**
	3	1.0763	0.8489
Gene expression + genetic mutation	1	1.0663	0.8503
	2	**0.9853**	**0.8616**
	3	1.1061	0.8447

Bold values represents the result is the best performance among the models participating in the comparison

**Table 3:** Performance of model training with different weight layers, self-attention, or *r*-radius

Weight layer	Self-attention	*r*-radius	MSE	$R^2$
Single	No	1	1.0848	0.8477
Single	No	2	**0.9804**	**0.8623**
Single	No	3	1.0694	0.8498
Multi	No	1	1.0654	0.8504
Multi	No	2	1.0078	0.8585
Multi	No	3	**0.9767**	**0.8628**
Multi	Yes	1	1.0724	0.8495
Multi	Yes	2	0.9785	0.8626
Multi	Yes	3	**0.9384**	**0.8683**
Multi	Yes	4	0.9917	0.8608
Multi	Yes	5	1.2083	0.8304

Bold values represents the result is the best performance among the models participating in the comparison

To study the significance of gene weight layer on model, we used the gene weight layer in Table 3 to combine the gene expression and genetic mutation. We tried applying the gene weight layer to the following: (1) genetic mutation only; (2) gene expression only; (3) genetic mutation and gene expression. Our model performed the best ($MSE = 0.9384$) when we applied the gene weight layer to genetic mutation only. Based on this experimental result, we finally decided to apply the gene weight layer to the genetic mutation only. In Table 3, when $r = 2$ radius, single weight layer without self-attention, the MSE is 0.9804. It is smaller than 0.9853, illustrating that through gene weight layer the two genetic traits are dynamically combined, achieving better performance than simple addition strategy. However, Table 3 shows that in single gene weight layer, $r = 2$, the MSE is 0.9804, bigger than 0.9727 in Table 2. This means the single weight layer has little side effects on model training. Taking genetic mutation data as input means more features are introduced into the model. Meanwhile, GNN is a deeper model, harder to train than CNN. This leads to the fact that during SWnet training, back propagation is more inclined to GNN network training, so as to obtain better MSE results. On the other hand, although our CNN gene branch added single weight layer, it has little effect on model training.

In fact, before training GNN as drug feature extract model, we have trained a model of SWnet with a similar structure. Its drug feature extraction is realized through the convolutional neural network and the input feature is fingerprints. When we did simple addition of the gene features, MSE was 1.0223. If we combined the gene features through gene weight layer, MSE was 0.9912. This indicates that the convolutional layer can make the genetic mutation participate in the training of the model well and improve the performance of the model. It is worth mentioning that although single weight layer has little effect on SWnet when drug branch is GNN, the MSE is 0.9804, better than CNN drug branch whose MSE is 0.9912.

### Multi-task and self-attention

In section “Single weight layer”, we have solved the problem of how to input both gene expression and genetic mutations through single gene weight layer. In this section, we discussed how to design the gene weight layer to balance the complexity between GNN and CNN. First, we introduced multi-task learning. The single gene weight layer was upgraded to multiple gene weight layer. Each drug corresponded to a single gene weight layer at a specific location. However, there are too many weight layers to train, resulting in the model’s predictive performance degradation. As shown in Table 3 when using multi-weight layers and $r = 3$ without self-attention, the MSE is 0.9911.

Then we introduced self-attention mechanism to improve gene branch of the model. With self-attention structure, drug similarity enhanced the training of multi-weight layers, and then gene expression and genetic mutation features can be better fused together. As shown in Table 3, when $r = 3$ and using multi-weight layers and self-attention, the $MSE = 0.9384$ and $R^2 = 0.8583$.

### Different *r*-radius

In section “Single weight layer” and section “Multi-task and self-attention” we have learned that the relative complexity of GNN and CNN determines the predictive ability of the model. With different *r*-radius subgraphs in GNN, we can adjust the complexity of the GNN branch. So we designed all experiments with different *r*-radius.

As shown in Table 3, single gene weight SWnet achieved the best performance when $r = 2$ radius, self-attention SWnet achieved the best performance when $r = 3$ radius. Compared the two model, since self-attention SWnet contained multiple matrix *W* and self-attention mechanism, it has more ability to extract molecule feature, therefore the GNN branch can be more complicated. Self-attention SWnet became overfitting when $r = 4$ radius. Meanwhile, single gene layer SWnet model is simpler than self-attention SWnet, it means that the model would become overfitting faster when $r = 3$ radius.

### Relationship between drug targets and genetic mutation status revealed by the gene weight layer

**Fig. 5:**
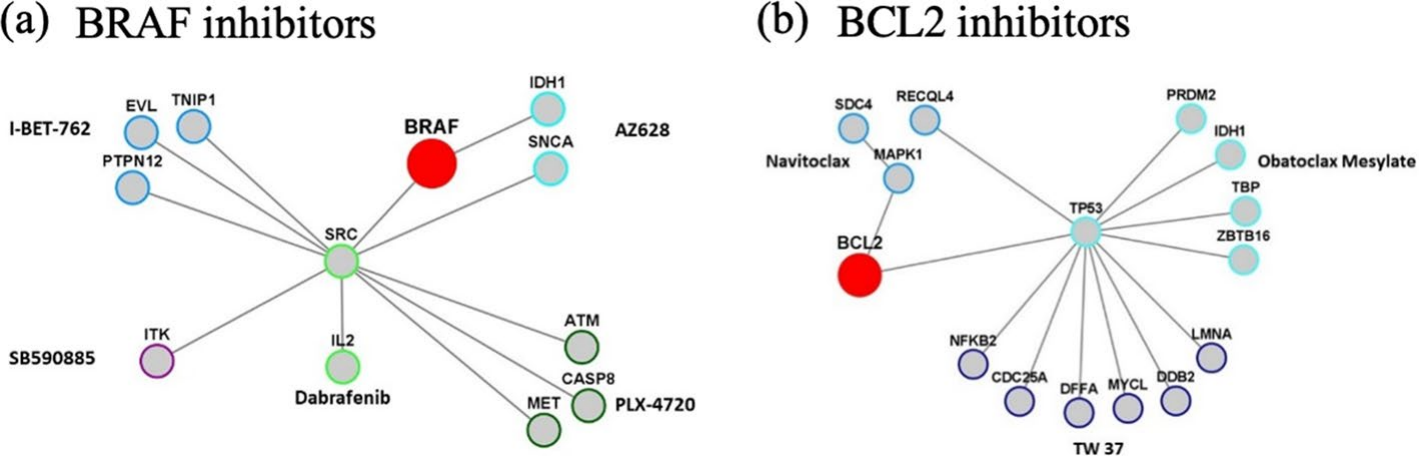
Physical interactions often occur among drug target proteins and proteins encoded by genes with the highest weight ($weight=1$). **a** Five BRAF inhibitors were included in the training data. Genes with $weight=1$ were extracted from the gene weight layer after model training, and their interactions with BRAF were analyzed in the String database. Connections within 2 degrees from BRAF were shown, grouped by drug. **b** Three BCL2 inhibitors was included in the training data. Analysis procedure was the same as above. The network showed connections within 2 degrees from BCL2

The trained gene weight layer reflected the normalized contribution of the binary genetic mutation status to the prediction of drug efficacy. To explore biological relevance of the model, genes with $weight=1$ have been identified for each drug, which represents the genetic mutation with the strongest predictive power. Proteins encoded by these genes might interact among themselves or interact with the drug targets. We identified these connections from protein–protein interaction database [39]. The protein–protein interactions were illustrated for BRAF inhibitors and BCL2 inhibitors (Fig. 5). Connections within 2 degrees from the target were shown in the network. For BRAF, two of the drugs (AZ628 and Dabrafenib) have their strongest predictive genes directly interacting with BRAF. For the other 3 BRAF inhibitors, there is at least one strongest predictive gene connected with BRAF within the second degree. The same was observed for BCL2 inhibitors. Interestingly, TP53 serves as a connection hub in the BCL2 case and SRC in the BRAF case. It is known that BCL2 and TP53 are two important nodes in the apoptosis signaling pathway and it has been reported that combining TP53 activation and BCL2 inhibition could result in synthetic lethality in AML [40]. Similarly, BRAF V600E mutation and SRC mutations have been found to be mutually exclusive in colorectal cancer patients, and both could serve as molecular markers for prognosis [41]. Lists of genes with $weight=1$ for each drug are available in Additional file 1: Table S1. These protein–protein interactions indicated the relevance of the gene weights in our model.

### Split training data with cell lines

To further explore the generalization ability of the model, we split the data set by cell lines, so that part of the genomic information never participated in the model training. We randomly divided training data with cell lines and trained GNN with different *r*-radius subgraphs, resulting in 159913 instances (90%) for training and 17215 instances (10%) for testing. As shown in Table 4, the MSE is 2.2793 when single weight layer with $r = 3$ radius. Since the cell lines are of different tissue lineages, the performance of the model is not as good as that when all the cell lines appeared in the training dataset. Meanwhile, when $r = 2$ and using multi-weight layers and self-attention, the $MSE = 2.2840$. This result is close to single layer SWnet. Due to weak cell line similarity, the different *r*-radius subgraphs have little effects of model performance.

**Table 4:** Performance of SWnet in terms of MSE in random split dataset by cell lines with different r-radius subgraphs

Weight layer	Self-attention	*r*-radius	MSE	$R^2$
Single	No	1	2.3090	0.6756
Single	No	2	2.3102	0.6755
Single	No	3	**2.2793**	**0.6799**
Multi	Yes	1	2.2975	0.6773
Multi	Yes	2	**2.2840**	**0.6792**
Multi	Yes	3	2.3304	0.6726

Bold values represents the result is the best performance among the models participating in the comparison

## Conclusion

In this paper, we proposed a new end-to-end deep learning model called SWnet. With the gene weight layer, we input the gene expression and genetic mutation at once. We used GNN to encode chemical structures of drugs with different *r*. We predicted the $IC_{50}$ based on gene signatures and molecular graphs and achieved better predictive performance than methods reported in previous literatures. We used the self-attention mechanism for the first time to explore the interactions between genetics and chemical structure of drugs.

SWnet combined genomic signatures and molecular graphs for drug efficacy prediction. We applied SWnet to GDSC and CCLE dataset, and showed that SWnet outperformed other models. With more datasets becoming available in the public domain, we envision training SWnet with more datasets and eventually using SWnet for in silico drug screenings. This model holds great promise for cancer therapy and precision medicine.

It is worth noting that the molecular structure information extracted by GNN is important for drug response prediction, where we applied the extend GNN to extract graph-level representation for molecule in this work. However, we used a simple arithmetic mean to get the graph-level representation from node-level representations. We believe that there will be more effective ways to extract the graph-level representation. We will improve the current GNN for more effective node information fusion in molecular graph.

The complex deep learning model SWnet can hardly be well-trained when it deals with a small dataset. In the future, we will design a GNN with a simpler structure to encode the drug structure to fit small datasets. We will try to fix the weight parameters for common atoms when updating node features in molecule. This may make GNN focus more on the structure of the molecule and the special atoms in it, and it will make it easier to train GNN with small datasets.

## Supplementary Information

**Additional file 1.** Drug and genes information for efficacy prediction.

## Data Availability

Source code can be downloaded from https://github.com/zuozhaorui/SWnet.git.

## Abbreviations

GDSC: Genomics of drug sensitivity in cancer
CCLE: Cancer cell line encyclopedia
GNN: Graph neural network
CNN: Convolutional neural network
SWnet: Self-attention gene weight layer network
SMILES: Simplified molecular input line entry system
$IC_{50}$: Half maximal inhibitory concentration
COSMIC: Catalogue of somatic mutations in cancer
MTL: Multi-task learning
MSE: Mean square error
KBMTL: Kernelized Bayesian multi-task learning
SRMF: Similarity-regularized matrix factorization
WGRMF: Weighted graph regularized matrix factorization
CDRscan: Cancer drug response profile scan
GraphDRP: Graph convolutional network for drug response prediction
