# Supplementary material for: SWnet: a deep learning model for drug response prediction from cancer genomic signatures and compound chemical structures
Source: BMC Bioinformatics. 2021 Sep 10;22:434. doi: 10.1186/s12859-021-04352-9 (PMC8434731; doi:10.1186/s12859-021-04352-9)
Supplement: Supplementary file 1 — Additional file 1. Drug and genes information for efficacy prediction. [file 12859_2021_4352_MOESM1_ESM.docx]

**Supplementary Table-1: Drug Information and corresponding genes with weight=1 for efficacy prediction**

| drug_id | drug_name | synonyms | pathway_name | targets | pubchem | gene_weight_1 | | |
| --- | --- | --- | --- | --- | --- | --- | --- | --- |
| 1013 | Nilotinib | Tasigna, AMN 107 | ABL signaling | ABL | 644241 | CBFB,ELK4,LAMA3,PFKL,RNPS1,TOP1,TRAPPC6A, | | |
| 34 | Imatinib | Gleevec, STI-571 | Other, kinases | ABL, KIT, PDGFR | 5291 | CNBD1,MELK, | | |
| 155 | Ponatinib | AP24534, AP-24534, KIN001-192, Iclusig | Other, kinases | ABL, PDGFRA, VEGFR2, FGFR1, SRC, TIE2, FLT3 | 24826799 | APP,ATRX,HADH,IPO13,NFKB2,WHSC1,ZMYM2, | | |
| 38 | Saracatinib | AZD0530, AZD-0530, AZ-10353926 | Other, kinases | ABL, SRC | 10302451 | HIST2H2BE,NCAPD2,PMAIP1, | | |
| 51 | Dasatinib | BMS-354825-03, BMS-354825, Sprycel | RTK signaling | ABL, SRC, Ephrins, PDGFR, KIT | 3062316 | ERO1A,FGFR1,GOLGA5,GPC3,KDM5C,MSI2,MTHFD2,NONO,POLG,PTGS2,RALB,TCEA1,TRIB3,UFM1, | | |
| 1053 | MK-2206 | MK 2206, MK2206 | PI3K/MTOR signaling | AKT1, AKT2 | 46930998 | BAG3,CCNA1,FOXA1,IDH2,PEX11A,PIH1D1,PLK1,RANBP2,RASA1,SERPINE1,SUPV3L1,TBC1D9B, | | |
| 228 | AKT inhibitor VIII | Akti-1/2, KIN001-102 | PI3K/MTOR signaling | AKT1, AKT2, AKT3 | 10196499 | CALR,ELL,H2AFV,MAP2K2,MUC4,NCOA3,PGM1,RRP8, | | |
| 86 | A-443654 | KIN001-139 | PI3K/MTOR signaling | AKT1, AKT2, AKT3 | 10172943 | CREB3L2,FNBP1,PCCB,PRPF4,SRGAP3,TCF7L2, | | |
| 326 | GSK690693 | GSK 690693, GSK-690693 | PI3K/MTOR signaling | AKT1, AKT2, AKT3 | 16725726 | BRCA1,DENND2D,FAM135B,GPATCH8,MTFR1,NCOA2,PACSIN3,ST6GALNAC2, | | |
| 171 | AKT inhibitor VIII | Akti-1/2, KIN001-102 | PI3K/MTOR signaling | AKT1, AKT2, AKT3 | 10196499 | BCL9L,FGFR2,FGFR3,HIP1,HIST1H2BK,LMO1,LZTR1,PECR,PSMD10,RELB,SFPQ,SMC4, | | |
| 35 | NVP-TAE684 | NVP-TAE 684, TAE684, TAE-684 | RTK signaling | ALK | 16038120 | CNOT4,GNA15,MB21D2,MLLT4,PACSIN3,PIH1D1,SCRN1, | | |
| 281 | Alectinib | CH5424802, CH 542802, Alecensa | RTK signaling | ALK | 49806720 | FHL2,FLT3,MMP1,PMM2,RASA1,SPP1,TESK1, | | |
| 304 | SB52334 | SB-52334, SB 52334 | Other, kinases | ALK5 | 9967941 | AKR7A2,ARNT,AURKB,NRIP1,STAT3,WT1, | | |
| 1001 | AICA Ribonucleotide | AICAR, N1-(b-D-Ribofuranosyl)-5-aminoimidazole-4-carboxamide | Metabolism | AMPK agonist | 65110 | JUN,NFATC2,PSIP1,SCYL3,SUFU,TICAM1, | | |
| 1072 | Avagacestat | BMS-708163, BMS 708163 | Other | Amyloid beta20, Amyloid beta40 | 46883536 | ADGRE5,BCOR,BCR,EIF3E,HACD3,PIN1,PTCH1,RALB,SNCA, | | |
| 205 | Avagacestat | BMS-708163, BMS 708163 | Other | Amyloid beta20, Amyloid beta40 | 46883536 | B4GAT1,HMOX1,KIT,RUVBL1,SLC25A46,TAL1,UBR7, | | |
| 286 | KIN001-236 | - | RTK signaling | Angiopoietin-1 receptor | none | SNAP25,SYNGR3, | | |
| 133 | Doxorubicin | Doxil, Rubex, Adriamycin, Adriablastin, Doxorubicine | DNA replication | Anthracycline | 31703 | BAD,GNAI1,GPC1,PIN1,RAD21,SMAD4, | | |
| 1006 | Cytarabine | Ara-Cytidine, Arabinosyl Cytosine, U-19920 | Other | Antimetabolite | 6253 | BIRC2,CDH10,COL2A1,FBLN2,FBXL12,GRB7,GRM3,HDAC2,MCM3,SS18,TGFBR2, | | |
| 1008 | Methotrexate | Abitrexate, Amethopterin, Rheumatrex, Trexall, Folex | DNA replication | Antimetabolite | 126941 | ANXA7,CCDC86,LSM6,MVP,PHGDH,PTEN,PWP1,RFC2,SYPL1, | | |
| 179 | 5-Fluorouracil | 5-FU | Other | Antimetabolite (DNA & RNA) | 3385 | B4GAT1,BPHL,CCR7,NUP93,RASA1, | | |
| 1502 | Bicalutamide | ICI-176334, Casodex, Cosudex, ICI 176334 | Hormone-related | AR | 2375 | BCOR,BIRC3,CHST11,CIAPIN1,CTSL,DYRK3,FANCF,G3BP1,MUC1,PIK3R3,PMM2,PTPRF,SOCS2,TOMM70,TPR,TRAF7, | | |
| 150 | Bicalutamide | ICI-176334, Casodex, Cosudex, ICI 176334 | Hormone-related | AR | 2375 | CDX2,CPEB3,FAIM,KDM6A,MEF2C,MLF1,MSI2,PCBP1,SMAD4,STXBP1,TRAPPC3,TUBB6,USP6, | | |
| 151 | QS11 | 944328-88-5 | Other | ARFGAP1 | 4263900 | EPHA7,LYRM1,MYBL2,RAE1,TCEAL4,WASF3,XPO7, | | |
| 152 | CP466722 | CP-466722, CP 466722, 1080622-86-1 | Genome integrity | ATM | 44551660 | ARHGAP26,AURKB,CNOT3,CSF1R,DNAJC15,KAT6A,MECOM,PBRM1,SETD1B,THAP11, | | |
| 1030 | KU-55933 | KU55933 | Genome integrity | ATM | 5278396 | CCNB1IP1,CRK,DNM1,GAA,HIST2H2BE,MEST,NUCB2,PPARG,PRPF40B,RHOH, | | |
| 225 | Genentech Cpd 10 | - | Mitosis | AURKA, AURKB | none | ACSL6,CDH1,EXT2,HMGCS1,MOK,MTOR,PDLIM1,RBM6,SNX13,TMCO1, | | |
| 226 | GSK1070916 | GSK-1070916 | Mitosis | AURKA, AURKC | 46885626 | CYLD,HEBP1,PTK2,RAI14,SIX1,SRC, | | |
| 258 | STF-62247 | STF62247 | Other | Autophagy inducer | 704473 | CLP1,COPB2,CRTAP,CUX1,SETD2,SOX21, | | |
| 1011 | Navitoclax | ABT-263, ABT263, ABT 263 | Apoptosis regulation | BCL2, BCL-XL, BCL-W | 24978538 | AKAP8L,ETFB,HSD17B10,MAPK1,NNT,RECQL4,SDC4, | | |
| 182 | Obatoclax Mesylate | GX15-070MS, Obatoclax, GX15-070 | Apoptosis regulation | BCL2, BCL-XL, BCL-W, MCL1 | 11404337 | HACD3,HS2ST1,IDH1,NUMA1,PRDM2,TBP,TP53,TRAM2,ZBTB16, | | |
| 1149 | TW 37 | TW37, TW-37 | Apoptosis regulation | BCL2, BCL-XL, MCL1 | 11455910 | ALDH7A1,CDC25A,DDB2,DFFA,ECT2L,HLA-DRA,LMNA,MYCL,NFKB2,PIGB,RNH1,TAL2,TMED10, | | |
| 52 | GNF-2 | KIN001-013 | ABL signaling | BCR-ABL | 5311510 | CSNK1E,CTNND2,CYB561,EPN2,GLI1,LRPAP1,NDRG1,NIT1,SRC,TBC1D31,USP22, | | |
| 196 | Phenformin | DBI | Other | Biguanide agent | 8249 | BCL10,BPHL,CCNF,CREB3L1,PRKAR1A, | | |
| 268 | Sepantronium bromide | YM155, YM-155, YM 155 | Apoptosis regulation | BIRC5 | 11178236 | EPB41L2,HNF1A,MDM2,MYD88,MYO5A,NSD1,OMD,YTHDF1,ZNF589,ZRSR2 | | |
| 1203 | QL-XII-61 | - | Other, kinases | BMX, BTK | none | CRTC3,MAPK1IP1L,PPARG, | | |
| 159 | HG6-64-1 | KIN001-206 | ERK MAPK signaling | BRAF | 53302361 | BACE2,DUSP22,EVL,FAIM,HMGA1,MAPK13,PTPN12,TNIP1, | | |
| 1036 | PLX-4720 | PLX4720, PLX 4720 | ERK MAPK signaling | BRAF | 24180719 | CCDC92,DROSHA,IL7R,KIF5C,MCM3,PTPRC,SRGAP3, | | |
| 29 | AZ628 | AZ-628, AZ 628 | ERK MAPK signaling | BRAF | 11676786 | CSNK1A1,IDH1,PAX8,SNCA,TAL2, | | |
| 1061 | SB590885 | SB-590885 | ERK MAPK signaling | BRAF | 11316960 | GTF2A2,ITK,S100A13,SH3BP5,TCEA2, | | |
| 1373 | Dabrafenib | GSK2118436, Tafinlar | ERK MAPK signaling | BRAF | 44462760 | CASP10,CFLAR,FPGS,IL2,MOK,RGS7,SRC,ST3GAL5,ST6GALNAC2,TRRAP,USP6NL, | | |
| 1371 | PLX-4720 | PLX4720, PLX 4720 | ERK MAPK signaling | BRAF | 24180719 | ATM,CASP8,DAXX,FLI1,GATA3,ICAM3,MET,TOR1A, | | |
| 275 | I-BET-762 | GSK525762A | Chromatin other | BRD2, BRD3, BRD4 | 46943432 | EVL,LRIG3,MACF1,PDIA5,SCYL3,SLC5A6,USP6NL, | | |
| 163 | JQ1 | JQ-1, (+)-JQ-1 | Chromatin other | BRD2, BRD3, BRD4, BRDT | 46907787 | ARHGEF10,BACE2,CSMD3,DCC,FES,IL1B,PLP2,PRKACA,TCF3,XBP1,YME1L1, | | |
| 1218 | JQ1 | JQ-1, (+)-JQ-1 | Chromatin other | BRD2, BRD3, BRD4, BRDT | 46907787 | EIF4EBP1,GABPB1,HMG20B,MALT1,POLB,SMARCE1,TXNRD1, | | |
| 1219 | PFI-1 | - | Chromatin other | BRD4 | 71271629 | FKBP14,HOXC11,KDM5C,MYCBP,NISCH,NPDC1,RGS2,RRP1B,USP7, | | |
| 192 | LFM-A13 | DDE-28 | Other, kinases | BTK | 54676905 | BCL3,BPHL,GRB7,IARS2,KAT6B,NR3C1,RPA1,TOP1,TP53BP2, | | |
| 331 | QL-X-138 | - | Other, kinases | BTK | none | AURKA,B2M,BCL6,CSNK1E,CTNND1,PAK6,POLG2,PYCR1,RAB4A,SKI,TRAM2,WASF3, | | |
| 235 | QL-XII-47 | - | Other, kinases | BTK, BMX | none | CYB561,EXT2,NUP98,PHGDH,PLOD3,PRKAR1A,PYGL,RFNG, | | |
| 1052 | RO-3306 | - | Cell cycle | CDK1 | 44450571 | CRLF2,DECR1,H3F3A,MAP7,MYO5A,PCK2,RFWD3,SMARCE1,TCF7L2,WHSC1L1,ZNF384, | | |
| 219 | AT-7519 | AT7519 | Cell cycle | CDK1, CDK2, CDK4, CDK6, CDK9 | 11338033 | BMP5,DRAP1,EML3,GAA,LRP10,NUMA1,PRAF2,TICAM1,TOR1A, | | |
| 53 | CGP-60474 | KIN001-019, CGP60474, CGP 60474 | Cell cycle | CDK1,CDK2,CDK5,CDK7,CDK9, PKC | 644215 | HLA-A,IL2,KNSTRN,NSDHL,PLEKHJ1,RAB21,RPL39L,TBPL1,VAT1,WIF1, | | |
| 301 | PHA-793887 | PHA793887, PHA 793887 | Cell cycle | CDK2, CDK7, CDK5 | 46191454 | BCL3,BCL7B,ITFG1,MDM2,ZNF586, | | |
| 110 | Seliciclib | Roscovitine, CYC-202, AL-39256 | Cell cycle | CDK2, CDK7, CDK9 | 160355 | ACLY,AFF3,GLI2,IL7R,INPP1,SBDS,TBL1XR1,TRIM33,ZNF589, | | |
| 54 | CGP-082996 | CINK4, KIN001-021 | Cell cycle | CDK4 | 24825971 | B4GAT1,CHD4,FAIM,NOLC1, | | |
| 1054 | Palbociclib | PD0332991, PD-0332991, PF-00080665-73 | Cell cycle | CDK4, CDK6 | 5330286 | CCNC,ELN,JAK1,MYH11,NSDHL,NUP214,PRCC,RHOA,TAL1,TNFRSF21,VAPB, | | |
| 346 | THZ-2-102-1 | - | Cell cycle | CDK7 | none | ATF5,CNBD1,CYLD,ELN,PTPRC,S100A7,TPR,TSPAN4, | | |
| 344 | THZ-2-49 | - | Cell cycle | CDK9 | none | AXIN1,CCL2,EPHB2,IPO13,TNC,TOR1A, | | |
| 345 | KIN001-270 | CDK9 inhibitor, CDK9-IN-1 | Cell cycle | CDK9 | 66577006 | CBLC,GLI2,PDS5A,PICALM,PPIE,TBC1D31,TIMM17B,UBE2L6, | | |
| 1022 | AZD7762 | AZD-7762, AZD 7762 | Cell cycle | CHEK1, CHEK2 | 11152667 | APP,DCTD,FGFR2,KLHL21,PHKB,SMARCC1,TXLNA,VPS72,WHSC1L1, | | |
| 1020 | Lenalidomide | CDC-501, CC-5013, Revlimid | Protein stability and degradation | CRBN | 216326 | COX6C,FCHO1,GLRX,LBR,NFKBIB,PLEKHJ1,RHEB,STAG1,STRN,VAV1, | | |
| 193 | GW-2580 | GX2580, CFMS receptor tyrosine kinase inhibitor | RTK signaling | CSF1R | 11617559 | CDH3,CHST11,CNTRL,FAM135B,IER3,KLHDC2,LEF1,NTRK1,PDHX,TET1, | | |
| 199 | Pazopanib | Votrient | RTK signaling | CSF1R, KIT, PDGFRA, PDGFRB | 10113978 | ATF1,CCR7,CHN1,CTNNA2,ELAVL1,ERBB3,ERCC3,MAPK9,SNX7,TSC1,ZNF384, | | |
| 329 | QL-XI-92 | - | Cytoskeleton | DDR1 | none | CAMSAP2,CDC42,COG2,PCMT1,SCRN1,TLE1,TOMM34, | | |
| 71 | Pyrimethamine | Daraprim, Chloridine | Other | Dihydrofolate reductase (DHFR) | 4993 | CHN1,EZH2,MUC16,NOL3,RPN1,SLC2A6,SLC37A4,SPDEF,TP53BP1,TXNDC9, | | |
| 1005 | Cisplatin | cis-Diammineplatinum(II) dichloride, Platinol, CIS-DDP | DNA replication | DNA crosslinker | 84691 | BAD,BCORL1,CCDC6,CIITA,KDM6A,NBN,NUP214,SMC1A,SSBP2, | | |
| 136 | Mitomycin-C | Mytozytrex, NSC-26980, MMC, Mitosol, Mitozytrex | DNA replication | DNA crosslinker | 5746 | CAST,EML3,KDM3A,PRCP,RARA,RHEB,SLC35B1, | | |
| 1038 | NU7441 | KU-57788, NU-7432, NU-7741 | Genome integrity | DNAPK | 11327430 | COG7,HIST2H2BE,MEST,PER1,SFN,SMARCD1, | | |
| 1264 | SGC0946 | - | Chromatin histone methylation | DOT1L | 56962337 | ARPP19,FANCG,HSPA4,MAP2K1,NCOA1,PDE4DIP,PIK3R3, | | |
| 1378 | Bleomycin (50 uM) | - | DNA replication | dsDNA break induction | 5460769 | AXIN1,CLP1,CTCF,HMOX1,PLEKHJ1,SCRN1,TFG,ZNF274, | | |
| 190 | Bleomycin | - | DNA replication | dsDNA break induction | several | ACD,AKT3,EZR,GADD45A,SBDS, | | |
| 1 | Erlotinib | Tarceva, RG-1415, CP-358774, OSI-774, Ro-508231, R-1415 | EGFR signaling | EGFR | 176870 | CTSL,MSN,PACSIN3,RET,SLC5A6,TBX3,TPM4,TXNRD1, | | |
| 1114 | Cetuximab | Erbitux, IMC-C225, C225, IMC-225, L01XC06 | EGFR signaling | EGFR | 85668777 | AKT2,BAZ1A,CISD1,DDB2,DHDDS,ECD,EIF4EBP1,ETS1,NIN,PCK2,PIGB,POT1,PTPN1,ROS1,SCCPDH, | | |
| 282 | Pelitinib | EKB-569, EKB 569 | EGFR signaling | EGFR | 6445562 | CAMTA1,DCAF12L2,EIF5,MAPKAPK5,PEX11A, | | |
| 1010 | Gefitinib | ZD-1839, Iressa | EGFR signaling | EGFR | 123631 | BAZ1A,BCL3,BCL7A,CHEK2,FEV,HACD3,MEF2C,PSMF1,RANBP2,RSPO2,SLC27A3,TPD52L2, | | |
| 1143 | HG-5-88-01 | - | Other, kinases | EGFR, ADCK4 | none | CD320,CRKL,KIF5C,PRPF4,PTPN13,RFX5,SOCS2,TLK2, | | |
| 119 | Lapatinib | Tykerb, Tyverb | RTK signaling | EGFR, ERBB2 | 208908 | BARD1,HOXA13,MUC16,PRAF2,TCF3,TLK2, | | |
| 1230 | IOX2 | IOX-2, IOX 2, AK176060 | Other | EGLN1 | 54685215 | ARHGEF2,CDC20,ETV6,MET,NCOA2,RBM15,SMARCA4, | | |
| 111 | Salubrinal | EIF-2alpha Inhibitor | Other | EIF2A | 5717801 | CLTC,MYH9,NPDC1,RABEP1, | | |
| 295 | NVP-BHG712 | BHG712 | RTK signaling | EPHB4 | 16747388 | DFFA,DNAJB1,PER1,SCCPDH,UTP14A, | | |
| 255 | CP724714 | CP-724714 | RTK signaling | ERBB2 | 9874913 | ARAF,ATP2B3,CTNND2,KDM3A,MRPS16,MSN,PXN,SLC35F2, | | |
| 1032 | Afatinib | BIBW2992, Tovok, Gilotrif | EGFR signaling | ERBB2, EGFR | 10184653 | BIRC2,CDH1,GTF2A2,NBEA,PAFAH1B3,SYPL1, | | |
| 1377 | Afatinib | BIBW2992, Tovok, Gilotrif | EGFR signaling | ERBB2, EGFR | 10184653 | BRIP1,CALU,CARS,CYLD,DMTF1,HK1,IL1B,IL2,ILK,NFATC4,PIK3CB,PRKCQ,SCARB1,ST7,TIMM17B,TMEM97, | | |
| 263 | FR-180204 | FR 180204, FR180204, ERK Inhibitor II | ERK MAPK signaling | ERK1, ERK2 | 11493598 | ACAT2,CDC73,CHEK2,HSPD1,ID2,LPP,TESK1,VTI1A, | | |
| 158 | PF-562271 | PF-00562271 | Cytoskeleton | FAK, FAK2 | 11713159 | CUL3,EGFR,ELAVL1,NCOR1,NR2F6,NT5C2,SYNGR3,TNIP1, | | |
| 166 | FTI-277 | - | Other | Farnesyl-transferase (FNTA) | 3005532 | ARID1A,ASAH1,BDH1,CASP3,CDH3,CRTAP,INPP4B,POLE,PPIC,PTPN12,TNIP1,TXNL4B,WDR7, | | |
| 1049 | PD173074 | PD-173074, PD 173074 | RTK signaling | FGFR1, FGFR2, FGFR3 | 1401 | ALDOA,ATP1A1,CDCA4,CSNK1A1,KIF14,TUBB6,WDR7, | | |
| 254 | Quizartinib | AC220, AC 220, AC-220, Asp-2689 | RTK signaling | FLT3 | 24889392 | CBFB,ERBB4,FH,FLNA,GALE,IL1B,LAGE3,LAP3,NPC1,NUP214,PAFAH1B1,PHGDH,RNMT, | | |
| 1024 | Lestaurtinib | CEP-701, SP-924, SPM-924, A-154475, KT-555 | Other, kinases | FLT3, JAK2, NTRK1, NTRK2, NTRK3 | 126565 | C5,KLK2,LIFR,LSM5,MYCL,ST3GAL5,SUPV3L1,TGFBR2,TP53BP2,TRAM2,TSPAN3, | | |
| 154 | CHIR-99021 | CT 99021, CHIR99021, CHIR 99021 | WNT signaling | GSK3A, GSK3B | 9956119 | ARHGEF10,DDX3X,HMGN2P46,MACF1,MVP,ZRSR2 | | |
| 1241 | CHIR-99021 | CT 99021, CHIR99021, CHIR 99021 | WNT signaling | GSK3A, GSK3B | 9956119 | AKAP8L,FH,FLNA,GRB10,JADE2,KIF5B,OXCT1,TBL1XR1,TCL1A,TMEM109, | | |
| 1025 | SB216763 | SB-216763, SB 216763 | WNT signaling | GSK3A, GSK3B | 176158 | CAT,CCNE2,DDB2,DUSP6,GLI2,GLRX,KDSR,NUMA1,PDIA5,PLK1,PSMG1,SLC5A6,TFDP1, | | |
| 200 | Dacinostat | NVP-LAQ824, LAQ824 | Chromatin histone acetylation | HDAC1 | 6445533 | ARHGEF10L,BAMBI,CCNF,DEK,EGF,ERC1,LSM14A,POLG2,RAB4A,RRAGA,SLC35A1,TPM1,ZDHHC6, | | |
| 272 | AR-42 | HDAC-42, AR 42, AR42 | Chromatin histone acetylation | HDAC1 | 6918848 | FBXO7,FCGR2B,IDH1,MYL9,NPC1, | | |
| 89 | Parthenolide | - | Chromatin histone acetylation | HDAC1 | 7251185 | COL2A1,CREG1,DFFB,FANCC,FOS,INTS3,MAP2K5,NIN,RHOH,RPL39L,TOMM70, | | |
| 274 | Belinostat | PXD101, PXD-101 | Chromatin histone acetylation | HDAC1 | 6918638 | ACVR1,CEP89,CREG1,FANCC,NET1,PNP,PPP1R13B,WIF1,YME1L1, | | |
| 164 | JQ12 | - | Chromatin histone acetylation | HDAC1, HDAC2 | none | DICER1,GNPDA1,KLF4,MAP4K4,MAPKAPK3,TERF2IP, | | |
| 88 | Entinostat | MS-275 | Chromatin histone acetylation | HDAC1, HDAC3 | 4261 | ARID4B,CDC73,CEBPZ,MBNL2,PDGFRA,SMC1A,UBR5,WFS1, | | |
| 276 | CAY10603 | - | Chromatin histone acetylation | HDAC1, HDAC6 | 24951314 | APP,AXIN2,CSF1R,ECT2L,JMJD6,LEF1,MTF2,NPDC1,RAB31,SMAD4,STIL,WASF3, | | |
| 273 | CUDC-101 | CUDC 101 | Other | HDAC1-10, EGFR, ERBB2 | 24756910 | DROSHA,DUSP3,HTATSF1,LMO2,MKL1,STAP2,STAT1, | | |
| 165 | DMOG | Dimethyloxalylglcine | Metabolism | HIF-PH | 560326 | B2M,BLM,CBFB,CHAC1,CORO1A,EIF1AX,FHL2,IFNAR1,KLHL21,PIM1,RAI14,SKI,TP63, | | |
| 1031 | Elesclomol | STA-4783 | Protein stability and degradation | HSP90 | 300471 | BACE2,CTNND1,JUN,MLF1,PCMT1,RHOH,SFN, | | |
| 1170 | CCT-018159 | CCT018159, CCT 018159 | Protein stability and degradation | HSP90 | 5327091 | CBR3,CREB1,PRCP,PRKCB,RABEP1,RTN2,TCTN1,WWTR1, | | |
| 328 | SNX-2112 | SNX 2112 | Protein stability and degradation | HSP90 | 24772860 | ARHGEF10L,ATRX,BARD1,GADD45B,MSRA,MYCN,PALB2,SNX11,SS18,TET2,TIMM22,TP63, | | |
| 194 | Luminespib | AUY922, VER-52296,NVP-AUY922, AUY | Protein stability and degradation | HSP90 | 10096043 | ATP1B1,ELL,ESR1,ORC1,PCMT1,PPIC,SORBS3,TIPARP, | | |
| 62 | BMS-536924 | BMS 536924 | IGF1R signaling | IGF1R, IR | 10390396 | ACLY,BCL2,COPB2,CRK,GNAI1,KAT7,POLE,PSMD10,SBDS, | | |
| 184 | BMS-754807 | BMS754807, BMS 754807 | RTK signaling | IGF1R, IR | 24785538 | ABL1,CFLAR,DNMT3A,EVL,NNT,NUP214,PALB2,RRP8,SACM1L, | | |
| 202 | GSK1904529A | GSK-1904529A, GSK 1904529A | RTK signaling | IGF1R, IR | 25124816 | BCOR,CCNE2,DUSP3,DYRK3,KMT2C,MUC4,P2RY8,PHKG2,PLOD3,PRKAG2,SH3GL1,TP53, | | |
| 1091 | BMS-536924 | BMS 536924 | IGF1R signaling | IGF1R, IR | 10390396 | CD44,COG2,FAS,PRKX,TCF3, | | |
| 91 | GSK319347A | KIN001-135, IKK-3 inhibitor | Other, kinases | IKK | 11626927 | TFEB,TRIB1, | | |
| 203 | BMS-345541 | BMS345541, IKK Inhibitor 3 | Other, kinases | IKK1, IKK2 | 9813758 | CASP3,KIAA0753,LMO1,NCKIPSD,SLC35B1,SMARCC1,TBPL1,TRIB1, | | |
| 305 | TPCA-1 | - | Other, kinases | IKK2 | 9903786 | CERK,CPNE3,FOXL2,H3F3A,MYCL,PDHX,PRDM16, | | |
| 290 | KIN001-260 | Bayer IKKb inhibitor, ACHP | Other, kinases | IKKB | 10451420 | AURKB,BCL3,BLM,CPNE3,EPN2,FANCF,GLOD4,KRAS,LMO2,NAB2,POLG2,SLC1A4,TET2,TXNDC9, | | |
| 1243 | Piperlongumine | Piplartine | Other | Induces reactive oxygen species | 637858 | CRTC1,DNAJC15,EPB41L2,PPIE,RHOA,TIMM22,TLR4, | | |
| 63 | BMS-509744 | KIN001-127, ITK inhibitor | Other, kinases | ITK | 20635522 | CTNND1,EPRS,EWSR1,FLI1,FOXO3,KDM6A,MFSD10,MLLT11,RRP8, | | |
| 206 | Ruxolitinib | INCB-18424, Ruxolitinib Phosphate, Jakafi | Other, kinases | JAK1, JAK2 | 25126798 | AKAP8L,APOE,ASXL1,FOXO4,FZD1,KTN1,LMO2,NBEA,PRR7,TCTA, | | |
| 306 | Fedratinib | TG101348, TG-101348, SAR302503, SAR-302503 | Other, kinases | JAK2 | 16722836 | ARHGAP1,ASPSCR1,BLMH,CDH17,CSNK1A1,CYP2C8,FAT3,ITGB1BP1,P4HA2,PMAIP1,PMS1,POP4,PTK2,TIMM17B,TLX1, | | |
| 1043 | JNK Inhibitor VIII | - | JNK and p38 signaling | JNK | 11624601 | CCNA1,DMTF1,SMC1A, | | |
| 207 | AS601245 | - | JNK and p38 signaling | JNK1, JNK2, JNK2 | 10109823 | A1CF,FZD1,MACF1,NF2,PTPRF,ZCCHC8, | | |
| 157 | JNK-9L | KIN001-204, JNK inhibitor 9l | JNK and p38 signaling | JNK2, JNK3 | 25222038 | ABHD6,ACBD3,ASXL2,CCL2,CCND3,FOXP1,GATA2,KDM3A,SCRN1,VAV1, | | |
| 41 | S-Trityl-L-cysteine | NSC 83265, Tritylcysteine | Mitosis | KIF11 | 76044 | APPBP2,BMPR1A,CIC,CIITA,FDFT1,FOSL1,FUBP1,PDHX,PWP1,SKI,TRRAP,WIPF2, | | |
| 298 | OSI-930 | OSI 930 OSI930 | RTK signaling | KIT | 9868037 | CIAPIN1,CSRP1,HOXC13,MECOM,PAK6,PCNA,PNP,PWWP2A,PYGL,RMI2,USP44,VAV3, | | |
| 293 | Amuvatinib | MP470, MP 470, MP-470 | RTK signaling | KIT, PDGFRA, FLT3 | 11282283 | BID,CD44,DCAF12L2,HLA-DRA,LIFR,MED12,PLAG1,RARA,RRP8,SHC1,STAT6,TRIB3,ZCCHC8, | | |
| 292 | Masitinib | AB1010, Masivet | RTK signaling | KIT, PDGFRA, PDGFRB | 10074640 | CETN3,NNT,SLC27A3,TBXA2R, | | |
| 208 | Ispinesib Mesylate | SB-715992 | Mitosis | KSP | 6450816 | CHCHD7,DDR1,LCK,NIPSNAP1,PLEKHJ1,SPDEF, | | |
| 256 | JW-7-24-1 | - | Other, kinases | LCK | none | ABHD4,AKT1,TFRC,TOMM34,U2AF1, | | |
| 55 | A-770041 | KIN001-111 | Other, kinases | LCK, FYN | 9549184 | ADRB2,DDR2,ELAVL1,FHL2,GLRX,HEY1,HSP90AA1,ISX,KIAA1549,PML,PTK2,SUPV3L1, | | |
| 1142 | HG-5-113-01 | - | Other | LOK, LTK, TRCB, ABL(T315I) | none | MYLK,SIRT3,SRC,ZBTB16, | | |
| 333 | T0901317 | TO-901317, TO901317 | Other | LXR, FXR | 447912 | CARS,CCR4,DUSP4,EGR1,EML4,FBXL12,PAK4,PDGFRA,PROS1,RALA,SGK1,SOX4,SYNE2,TIMELESS, | | |
| 291 | KIN001-266 | - | ERK MAPK signaling | MAP3K8 | 44143370 | ATMIN,CIITA,EAPP,MDM2,MEF2C,PALB2,USP44, | | |
| 1133 | Serdemetan | JNJ-26854165 | p53 pathway | MDM2 | 11609586 | ATP6V0B,CCDC92,CHST11,DLD,ITFG1,KIF14,PARP1,ZNF131, | | |
| 1047 | Nutlin-3a (-) | - | p53 pathway | MDM2 | 11433190 | CSNK1A1,DERA,ITGB1BP1,LRP10,MALT1,MYLK,NSDHL, | | |
| 269 | NSC-207895 | XI-006, NSC207895 | p53 pathway | MDM4 | 42640 | CALU,CANT1,CHN1,CHP1,EBP,GLRX,MYO5A,NAB2,NCKIPSD,NFKBIA,PLP2,PTPN12,TIMM17B, | | |
| 1062 | Selumetinib | AZD6244, AZD-6244, ARRY-886 | ERK MAPK signaling | MEK1, MEK2 | 10127622 | DDX6,EGFR,KIF2C,NRAS,SKI,TBP,TBPL1,TP53,WAS, | | |
| 1498 | Selumetinib | AZD6244, AZD-6244, ARRY-886 | ERK MAPK signaling | MEK1, MEK2 | 10127622 | ALDH7A1,FOXA1,GRWD1,KCNK1,LSM14A,PACSIN3,PGRMC1,PSMF1,TICAM1, | | |
| 1060 | PD0325901 | PD-0325901, PD 0325901 | ERK MAPK signaling | MEK1, MEK2 | 9826528 | ATP2B3,BCLAF1,BDH1,CNDP2,HMGCR,IER3,KIF20A,MEF2C,NCOA1,NOSIP,SCYL3,TCFL5,VAV1,ZNF589, | | |
| 1014 | Refametinib | RDEA119, BAY-86-9766, BAY 869766 | ERK MAPK signaling | MEK1, MEK2 | 44182295 | CD40,EDN1,ERBB3,FEZ2,HOOK3,HOXD13,KLHL21,NAB2,NCAPD2,NFKBIB,PRR15L,SCAND1,XBP1,ZDHHC6, | | |
| 1015 | CI-1040 | CI 1040, PD-18435, PD-184352, 212631-79-3 | ERK MAPK signaling | MEK1, MEK2 | 6918454 | CHD4,EDN1,ENOSF1,IGF1R,MTHFD2,PLK1, | | |
| 1526 | Refametinib | RDEA119, BAY-86-9766, BAY 869766 | ERK MAPK signaling | MEK1, MEK2 | 44182295 | DNM1L,MOK,NTHL1,PPIC,SLC25A4,STAMBP,VAPB, | | |
| 279 | BIX02189 | BIX 02189 | ERK MAPK signaling | MEK5, ERK5 | 46931012 | CRTC1,FGFR3,HLA-DRA,MLLT4,SPRED2, | | |
| 6 | PHA-665752 | PHA665752, PHA 665752 | RTK signaling | MET | 10461815 | ACBD3,ARHGAP1,ARID1A,CBLB,CD40,FBXO21,GATA1,IL21R,NCOR2,PDHX,PTPRK,SOX21,TCL1A,WDR61, | | |
| 37 | Crizotinib | Xalkori, PF2341066, PF-2341066, PF 2341066 | RTK signaling | MET, ALK, ROS1 | 11626560 | BNIP3,CCDC86,GATA2,MCOLN1,NCKIPSD,NIN,NUP214,P4HA2,SIX1,ZNF451, | | |
| 308 | Foretinib | GSK1363089, XL-880, EXEL-2880, GSK089 | RTK signaling | MET, KDR, TIE2, VEGFR3/FLT4, RON, PDGFR, FGFR1, EGFR | 42642645 | ABHD4,ANK1,CREBBP,ELN,FOXL2,IGHMBP2,PDHX,RAB27A,SLC35A3,SLC35B1, | | |
| 1007 | Docetaxel | RP-56976, Taxotere | Mitosis | Microtubule stabiliser | 148124 | B4GAT1,DUSP3,FAS,HADH,ISX,KAT6B,KDM3A,PLEKHJ1,RANBP2,RFWD3,SOX4,TRIM27, | | |
| 11 | Paclitaxel | BMS-181339-01, Taxol, Onxol, Paxene, Praxel, Abraxane | Mitosis | Microtubule stabiliser | 36314 | CD209,ETV5,HMOX1,JAZF1,PRKCD,RSU1,SLC2A6,STAT3,TCTA, | | |
| 201 | Epothilone B | Patupilone, EpoB, EPO906, GNF-PF-193 | Mitosis | Microtubule stabiliser | 448013 | ARPP19,CALU,HSPA4,NBEA,PNP,RAC1,RFWD3,TBC1D9B,TNC,USP6NL,VTI1A,ZMIZ1, | | |
| 294 | MPS-1-IN-1 | - | Mitosis | MPS1 | 25195352 | ANXA7,ARID4B,CNBD1,CSRP1,ECH1,FPGS,PLEKHJ1,SLC35F2, | | |
| 83 | JW-7-52-1 | NA | PI3K/MTOR signaling | MTOR | 49836027 | B4GAT1,BIRC2,FZD7,HEATR1,PTPRK,RFWD3,SACM1L,SUZ12,TCF7L2,UGDH, | | |
| 1166 | QL-VIII-58 | - | Other | MTOR, ATR | none | CDK1,CSNK1E,ERCC3,ERG,FOXJ3,NBN,SOCS2,TFDP1, | | |
| 3 | Rapamycin | AY-22989, Sirolimus, WY-090217, Torisel, Rapamune | PI3K/MTOR signaling | MTORC1 | 5384616 | BID,C2CD5,CCDC86,RNMT, | | |
| 299 | OSI-027 | A-1065-5 | PI3K/MTOR signaling | MTORC1, MTORC2 | 44224160 | BRAF,CDC45,CTNND2,EPB41L2,FAT1,GAS7,HSPA4,MYCBP2,PAK6,SLC11A2, | | |
| 1059 | AZD8055 | AZD-8055 | PI3K/MTOR signaling | MTORC1, MTORC2 | 25262965 | B4GAT1,CARS,ERBB3,NCOA3,NIN,POT1, | | |
| 1529 | Pevonedistat | MLN4924, MLN 4924, MLN-4924 | Other | NAE | 16720766 | ATM,CHP1,MYO10,RAF1,SKP1, | | |
| 1248 | Daporinad | APO866, FK866, FK866 | Metabolism | NAMPT | 6914657 | FANCG,FOXO3,HMGA2,MDM2,MSH6,NTRK1,RMI2,SMARCD1, | | |
| 1266 | ICL1100013 | - | Other | N-myristoyltransferase 1/2 | - | ATP6V1D,BAMBI,CLTCL1,FAM135B,FNBP1,IGF2R,MAMLD1,MTFR1,NUP88,NUTM1,VAPB,ZNF131, | | |
| 211 | TL-2-105 | - | Other | not defined | none | CALR,FAT1,HLA-DRA,LARP4B,MN1,MTHFD2,NUP133,RAD51B,RFX5,VAV1, | | |
| 170 | Shikonin | Anchusin | Other | not defined | 5208 | FAM131B,FAM20B,GATA3,ISX,MELK,PCBP1,PDGFA,SLC2A6, | | |
| 1023 | GW441756 | GW 441756 | RTK signaling | NTRK1 | 9943465 | CNBD1,H2AFV,NTRK1,PCNA,PTPN13,TFAP2A, | | |
| 1042 | Doramapimod | BIRB-796, BIRB 796 | JNK and p38 signaling | p38, JNK2 | 156422 | FOXL2,HES1,IGF2R,TLX3, | | |
| 221 | TAK-715 | KIN001-201, TAK 715 | JNK and p38 signaling | p38alpha, p38beta | 9952773 | ARID4B,CDC25B,NCOA2,PXN,RFC5,SETD2,SH2B3,STX4,TBX2, | | |
| 176 | IPA-3 | IPA 3 | Cytoskeleton | PAK1 | 521106 | ARHGEF10L,HLF,HSPA4,IARS2,MLLT1,NRAS,TOMM70,TSC1,XPC, | | |
| 1175 | Rucaparib | PF-01367338, AG-014699, AG-14447, AG-14699 | Genome integrity | PARP1, PARP2 | 9931953 | ARID4B,CENPE,DCAF12L2,EXT1,FANCC,RFWD3,SBDS,SS18,TGFBR2,TM9SF2,WNK2, | | |
| 1259 | Talazoparib | BMN-673, BMN 973 | Genome integrity | PARP1, PARP2 | 44819241 | AGL,CCNE2,DAG1,DDX6,HS2ST1,LYRM1,PRKCB,PRPF4,TSPAN4, | | |
| 1017 | Olaparib | AZD2281, KU0059436, Lynparza | Genome integrity | PARP1, PARP2 | 23725625 | C2CD5,C5,EIF3E,GRWD1,IL1B,IL6ST,MAPK1,RAP1GDS1,SIX2,TP53, | | |
| 1495 | Olaparib | AZD2281, KU0059436, Lynparza | Genome integrity | PARP1, PARP2 | 23725625 | CPSF4,FBXW7,GLRX,HOMER2,ITGB1BP1,LMO1,PAK4,PPFIBP1,TP53BP1, | | |
| 5 | Sunitinib | Sutent, Sunitinib Malate, SU-11248 | RTK signaling | PDGFR, KIT, VEGFR, FLT3, RET, CSF1R | 5329102 | FLT4,FOXA1,LRIG3,MAMLD1,MIF,MLLT11,PDGFA,RFNG,RNMT,SGK1,SLC11A2,STRN, | | |
| 30 | Sorafenib | Nexavar, 284461-73-0, BAY 43-9006 | RTK signaling | PDGFR, KIT, VEGFR, RAF | 216239 | ACVR1,LARP4B,PRDM2,SCYL3,TMPRSS2, | | |
| 167 | OSU-03012 | AR-12, OSU 03012, OSU03012, PDK1 inhibitor AR-12 | Metabolism | PDK1 (PDPK1) | 10027278 | CASC5,COL2A1,DUSP11,FGFR4,HEY1,NET1,NUP133,OMD,PMAIP1,PTPRF,RABEP1,TUBB6, | | |
| 287 | KIN001-244 | PDK1 inhibitor 7 | Metabolism | PDK1 (PDPK1) | 56965967 | CANT1,CDC45,EP300,FLT3,HOXD13,PHF6,RAC1,RPN1,ZNF451, | | |
| 222 | BX-912 | - | Metabolism | PDK1 (PDPK1) | 11754511 | CASP7,CIRBP,CNPY3,LMNA,NCK2,PRRX1,PTK2B,RSU1,TRAPPC3,TXNRD1, | | |
| 1058 | Pictilisib | GDC-0941, GDC0941, RG-7621 | PI3K/MTOR signaling | PI3K (class 1) | 17755052 | CCNB1IP1,CXCL2,DLD,DNM2,GNA11,MUC16,PPP2R3C,QKI,SLC35F2,TBP,XBP1,ZNF429, | | |
| 1527 | Pictilisib | GDC-0941, GDC0941, RG-7621 | PI3K/MTOR signaling | PI3K (class 1) | 17755052 | CCNA1,CNOT4,GLI2,ICAM1,IKZF1,KIAA0753,MAP2K1,PHKA1,POT1,SUV39H1, | | |
| 1057 | Dactolisib | NVP-BEZ235, BEZ235 | PI3K/MTOR signaling | PI3K (class 1), MTORC1, MTORC2 | 11977753 | ACKR3,AKT3,ARPP19,CD79A,MECOM,PHGDH,PTPN6,RAB4A,SND1,TFEB,TSC22D3, | | |
| 283 | Omipalisib | GSK2126458, GSK-2126458, EX-8678, GSK458 | PI3K/MTOR signaling | PI3K (class 1), MTORC1, MTORC2 | 25167777 | BZW2,HMOX1,SETBP1,SMARCC1, | | |
| 302 | PI-103 | PI-103, PI103, PI 103 | Other, kinases | PI3Kalpha, DAPK3, CLK4, PIM3, HIPK2 | 9884685 | DROSHA,PARP2,PTK2B,SMARCC1,THAP11,TLK2, | | |
| 94 | TGX221 | TGX-221, Tgx 221 | PI3K/MTOR signaling | PI3Kbeta | 9907093 | BZW2,CCNF,FZD7,MSN,NUP93,TMEM109,TOMM34, | | |
| 1066 | AZD6482 | AZD 6482, AZD-6482, AK-55409 | PI3K/MTOR signaling | PI3Kbeta | 44137675 | ABI1,FEZ2,POT1,SMARCE1, | | |
| 156 | AZD6482 | AZD 6482, AZD-6482, AK-55409 | PI3K/MTOR signaling | PI3Kbeta | 44137675 | CHST11,FZD7,GNA11,NCK2,PSMG1,SPR,STAT5B,TFDP1, | | |
| 238 | Idelalisib | CAL-101, Zydelig | PI3K/MTOR signaling | PI3Kdelta | 11625818 | CTNNAL1,KRAS,ME2,MUTYH,NFATC3,PPP2R1A,TCFL5,TIAM1, | | |
| 224 | AS605240 | KIN001-173, AS-605240 | PI3K/MTOR signaling | PI3Kgamma | 5289247 | ARHGAP26,ARHGAP5,CSF3R,ELF4,GPC5,GRB7,IDH2,IQGAP1,KMT2D,MAN2B1,NDRG1,RAD51B,SLC5A6,SSX1,TFG, | | |
| 303 | PIK-93 | PIK 93, PIK93 | PI3K/MTOR signaling | PI3Kgamma | 6852167 | ARHGEF10,BDH1,LASP1,NR3C1,RAP1GAP,SNX11, | | |
| 197 | Bryostatin 1 | Bryostatin | Other | PKC | 5280757 | CASC3,POLD1, | | |
| 153 | Midostaurin | PKC412, benzoylstaurosporine, CGP-41251 | Other | PKC, PPK, FLT1, c-FGR, others | several | AMER1,ARID4B,CDH11,FBXL12,FOXJ3,HK1,HMGA1,MRPL19,NONO,PRDM1,USP8, | | |
| 229 | Enzastaurin | LY317615 | Other, kinases | PKCB | 176167 | CPEB3,DPH2,ECD,FSD1,GAA,HOXA11,HYOU1,IL2,KLHL21,LMO1,PAF1,ROBO2,TET1,TRIM24,TRIP11, | | |
| 87 | GW843682X | GW843682X (AN-13) | Cell cycle | PLK1 | 9826308 | CORO1A,DDX3X,ICMT,LBR,LPP,MSH6,NIPSNAP1,PAK1,PRKAG2,PRKX,RTN2,SH2B3,TBC1D9B,TCF7L2,TIMELESS, | | |
| 60 | BI-2536 | - | Cell cycle | PLK1, PLK2, PLK3 | 11364421 | AKT1,GRWD1,HLA-A,INPP4B,LYN,MBTPS1,NUP133,PDHX,PTPN1,TJP1, | | |
| 257 | NPK76-II-72-1 | - | Cell cycle | PLK3 | none | AFF4,ELAC2,HOOK3,MUC4,TERF2IP, | | |
| 173 | FH535 | - | Other | PPARgamma, PPARdelta | 3463933 | CETN3,ELF4,ERCC4,FCRL4,FGFR1,GNB5,ME2,MYB,NCKIPSD,PRRX1, | | |
| 1067 | CCT007093 | - | Cell cycle | PPM1D | 2314623 | LIFR,MUC1,NUP88,PTPN1,PTPRF,WWTR1, | | |
| 175 | PAC-1 | GTPL5238 | Apoptosis regulation | Procaspase-3, Procaspase-7 | 6753378 | ANK1,ASXL2,ATR,AURKA,BCL7A,DUSP4,LRRC41,MYC,SLC11A2,TRAK2,ZRSR2 | | |
| 104 | Bortezomib | PS-341, LDP-341, Velcade | Protein stability and degradation | Proteasome | 387447 | COX6C,FPGS,H2AFV,ITGB1BP1,LAP3,MLLT10,NUP133,PNKP,SLC37A4,SQSTM1,USP14,XPC, | | |
| 9 | MG-132 | LLL cpd, MG 132, MG132 | Protein stability and degradation | Proteasome, CAPN1 | 462382 | DNAJB2,ETNK1,RALB,TLX3,ZNF429, | | |
| 135 | Gemcitabine | Gemzar, LY-188011 | DNA replication | Pyrimidine antimetabolite | 60750 | CDC42,CDH11,CHEK2,CSNK1E,ELN,GLI1,GPER1,LBR,LHFP,LYRM1,PLK1,PTK2B,RGS2,SOX4,TES,TRIB3, | | |
| 1069 | EHT-1864 | EHT 1864 | Cytoskeleton | RAC1, RAC2, RAC3 | 9938202 | BCL3,ERCC4,LSM14A,MBOAT7,MDM2,NUDT9,PTCH1,RGS7,SLC35B1,TCF3,ZMIZ1, | | |
| 186 | Bexarotene | LG-100069, Targretin, Targret, Targrexin, Targretyn, Bexarotenum | Other | Retinioic X receptor (RXR) agonist | 82146 | EZH2,IL6ST,ITGB5,MYCBP2,SCRN1,SDHA,SFPQ, | | |
| 300 | CX-5461 | CX5461, CX 5461 | Other | RNA Polymerase 1 | 25257557 | ANO10,EPAS1,ETFB,EXT2,HMGA2,MALAT1,MBTPS1,NIT1,RNF167,TBPL1,TCEA1,TMEM109,VTI1A, | | |
| drug_id | drug_name | synonyms | pathway_name | targets | pubchem | | gene_weight_1 |  |
| 1013 | Nilotinib | Tasigna, AMN 107 | ABL signaling | ABL | 644241 | | CBFB,ELK4,LAMA3,PFKL,RNPS1,TOP1,TRAPPC6A, |  |
| 34 | Imatinib | Gleevec, STI-571 | Other, kinases | ABL, KIT, PDGFR | 5291 | | CNBD1,MELK, |  |
| 155 | Ponatinib | AP24534, AP-24534, KIN001-192, Iclusig | Other, kinases | ABL, PDGFRA, VEGFR2, FGFR1, SRC, TIE2, FLT3 | 24826799 | | APP,ATRX,HADH,IPO13,NFKB2,WHSC1,ZMYM2, |  |
| 38 | Saracatinib | AZD0530, AZD-0530, AZ-10353926 | Other, kinases | ABL, SRC | 10302451 | | HIST2H2BE,NCAPD2,PMAIP1, |  |
| 51 | Dasatinib | BMS-354825-03, BMS-354825, Sprycel | RTK signaling | ABL, SRC, Ephrins, PDGFR, KIT | 3062316 | | ERO1A,FGFR1,GOLGA5,GPC3,KDM5C,MSI2,MTHFD2,NONO,POLG,PTGS2,RALB,TCEA1,TRIB3,UFM1, |  |
| 1053 | MK-2206 | MK 2206, MK2206 | PI3K/MTOR signaling | AKT1, AKT2 | 46930998 | | BAG3,CCNA1,FOXA1,IDH2,PEX11A,PIH1D1,PLK1,RANBP2,RASA1,SERPINE1,SUPV3L1,TBC1D9B, |  |
| 228 | AKT inhibitor VIII | Akti-1/2, KIN001-102 | PI3K/MTOR signaling | AKT1, AKT2, AKT3 | 10196499 | | CALR,ELL,H2AFV,MAP2K2,MUC4,NCOA3,PGM1,RRP8, |  |
| 86 | A-443654 | KIN001-139 | PI3K/MTOR signaling | AKT1, AKT2, AKT3 | 10172943 | | CREB3L2,FNBP1,PCCB,PRPF4,SRGAP3,TCF7L2, |  |
| 326 | GSK690693 | GSK 690693, GSK-690693 | PI3K/MTOR signaling | AKT1, AKT2, AKT3 | 16725726 | | BRCA1,DENND2D,FAM135B,GPATCH8,MTFR1,NCOA2,PACSIN3,ST6GALNAC2, |  |
| 171 | AKT inhibitor VIII | Akti-1/2, KIN001-102 | PI3K/MTOR signaling | AKT1, AKT2, AKT3 | 10196499 | | BCL9L,FGFR2,FGFR3,HIP1,HIST1H2BK,LMO1,LZTR1,PECR,PSMD10,RELB,SFPQ,SMC4, |  |
| 35 | NVP-TAE684 | NVP-TAE 684, TAE684, TAE-684 | RTK signaling | ALK | 16038120 | | CNOT4,GNA15,MB21D2,MLLT4,PACSIN3,PIH1D1,SCRN1, |  |
| 281 | Alectinib | CH5424802, CH 542802, Alecensa | RTK signaling | ALK | 49806720 | | FHL2,FLT3,MMP1,PMM2,RASA1,SPP1,TESK1, |  |
| 304 | SB52334 | SB-52334, SB 52334 | Other, kinases | ALK5 | 9967941 | | AKR7A2,ARNT,AURKB,NRIP1,STAT3,WT1, |  |
| 1001 | AICA Ribonucleotide | AICAR, N1-(b-D-Ribofuranosyl)-5-aminoimidazole-4-carboxamide | Metabolism | AMPK agonist | 65110 | | JUN,NFATC2,PSIP1,SCYL3,SUFU,TICAM1, |  |
| 1072 | Avagacestat | BMS-708163, BMS 708163 | Other | Amyloid beta20, Amyloid beta40 | 46883536 | | ADGRE5,BCOR,BCR,EIF3E,HACD3,PIN1,PTCH1,RALB,SNCA, |  |
| 205 | Avagacestat | BMS-708163, BMS 708163 | Other | Amyloid beta20, Amyloid beta40 | 46883536 | | B4GAT1,HMOX1,KIT,RUVBL1,SLC25A46,TAL1,UBR7, |  |
| 286 | KIN001-236 | - | RTK signaling | Angiopoietin-1 receptor | none | | SNAP25,SYNGR3, |  |
| 133 | Doxorubicin | Doxil, Rubex, Adriamycin, Adriablastin, Doxorubicine | DNA replication | Anthracycline | 31703 | | BAD,GNAI1,GPC1,PIN1,RAD21,SMAD4, |  |
| 1006 | Cytarabine | Ara-Cytidine, Arabinosyl Cytosine, U-19920 | Other | Antimetabolite | 6253 | | BIRC2,CDH10,COL2A1,FBLN2,FBXL12,GRB7,GRM3,HDAC2,MCM3,SS18,TGFBR2, |  |
| 1008 | Methotrexate | Abitrexate, Amethopterin, Rheumatrex, Trexall, Folex | DNA replication | Antimetabolite | 126941 | | ANXA7,CCDC86,LSM6,MVP,PHGDH,PTEN,PWP1,RFC2,SYPL1, |  |
| 179 | 5-Fluorouracil | 5-FU | Other | Antimetabolite (DNA & RNA) | 3385 | | B4GAT1,BPHL,CCR7,NUP93,RASA1, |  |
| 1502 | Bicalutamide | ICI-176334, Casodex, Cosudex, ICI 176334 | Hormone-related | AR | 2375 | | BCOR,BIRC3,CHST11,CIAPIN1,CTSL,DYRK3,FANCF,G3BP1,MUC1,PIK3R3,PMM2,PTPRF,SOCS2,TOMM70,TPR,TRAF7, |  |
| 150 | Bicalutamide | ICI-176334, Casodex, Cosudex, ICI 176334 | Hormone-related | AR | 2375 | | CDX2,CPEB3,FAIM,KDM6A,MEF2C,MLF1,MSI2,PCBP1,SMAD4,STXBP1,TRAPPC3,TUBB6,USP6, |  |
| 151 | QS11 | 944328-88-5 | Other | ARFGAP1 | 4263900 | | EPHA7,LYRM1,MYBL2,RAE1,TCEAL4,WASF3,XPO7, |  |
| 152 | CP466722 | CP-466722, CP 466722, 1080622-86-1 | Genome integrity | ATM | 44551660 | | ARHGAP26,AURKB,CNOT3,CSF1R,DNAJC15,KAT6A,MECOM,PBRM1,SETD1B,THAP11, |  |
| 1030 | KU-55933 | KU55933 | Genome integrity | ATM | 5278396 | | CCNB1IP1,CRK,DNM1,GAA,HIST2H2BE,MEST,NUCB2,PPARG,PRPF40B,RHOH, |  |
| 225 | Genentech Cpd 10 | - | Mitosis | AURKA, AURKB | none | | ACSL6,CDH1,EXT2,HMGCS1,MOK,MTOR,PDLIM1,RBM6,SNX13,TMCO1, |  |
| 226 | GSK1070916 | GSK-1070916 | Mitosis | AURKA, AURKC | 46885626 | | CYLD,HEBP1,PTK2,RAI14,SIX1,SRC, |  |
| 258 | STF-62247 | STF62247 | Other | Autophagy inducer | 704473 | | CLP1,COPB2,CRTAP,CUX1,SETD2,SOX21, |  |
| 1011 | Navitoclax | ABT-263, ABT263, ABT 263 | Apoptosis regulation | BCL2, BCL-XL, BCL-W | 24978538 | | AKAP8L,ETFB,HSD17B10,MAPK1,NNT,RECQL4,SDC4, |  |
| 182 | Obatoclax Mesylate | GX15-070MS, Obatoclax, GX15-070 | Apoptosis regulation | BCL2, BCL-XL, BCL-W, MCL1 | 11404337 | | HACD3,HS2ST1,IDH1,NUMA1,PRDM2,TBP,TP53,TRAM2,ZBTB16, |  |
| 1149 | TW 37 | TW37, TW-37 | Apoptosis regulation | BCL2, BCL-XL, MCL1 | 11455910 | | ALDH7A1,CDC25A,DDB2,DFFA,ECT2L,HLA-DRA,LMNA,MYCL,NFKB2,PIGB,RNH1,TAL2,TMED10, |  |
| 52 | GNF-2 | KIN001-013 | ABL signaling | BCR-ABL | 5311510 | | CSNK1E,CTNND2,CYB561,EPN2,GLI1,LRPAP1,NDRG1,NIT1,SRC,TBC1D31,USP22, |  |
| 196 | Phenformin | DBI | Other | Biguanide agent | 8249 | | BCL10,BPHL,CCNF,CREB3L1,PRKAR1A, |  |
| 268 | Sepantronium bromide | YM155, YM-155, YM 155 | Apoptosis regulation | BIRC5 | 11178236 | | EPB41L2,HNF1A,MDM2,MYD88,MYO5A,NSD1,OMD,YTHDF1,ZNF589,ZRSR2 |  |
| 1203 | QL-XII-61 | - | Other, kinases | BMX, BTK | none | | CRTC3,MAPK1IP1L,PPARG, |  |
| 159 | HG6-64-1 | KIN001-206 | ERK MAPK signaling | BRAF | 53302361 | | BACE2,DUSP22,EVL,FAIM,HMGA1,MAPK13,PTPN12,TNIP1, |  |
| 1036 | PLX-4720 | PLX4720, PLX 4720 | ERK MAPK signaling | BRAF | 24180719 | | CCDC92,DROSHA,IL7R,KIF5C,MCM3,PTPRC,SRGAP3, |  |
| 29 | AZ628 | AZ-628, AZ 628 | ERK MAPK signaling | BRAF | 11676786 | | CSNK1A1,IDH1,PAX8,SNCA,TAL2, |  |
| 1061 | SB590885 | SB-590885 | ERK MAPK signaling | BRAF | 11316960 | | GTF2A2,ITK,S100A13,SH3BP5,TCEA2, |  |
| 1373 | Dabrafenib | GSK2118436, Tafinlar | ERK MAPK signaling | BRAF | 44462760 | | CASP10,CFLAR,FPGS,IL2,MOK,RGS7,SRC,ST3GAL5,ST6GALNAC2,TRRAP,USP6NL, |  |
| 1371 | PLX-4720 | PLX4720, PLX 4720 | ERK MAPK signaling | BRAF | 24180719 | | ATM,CASP8,DAXX,FLI1,GATA3,ICAM3,MET,TOR1A, |  |
| 275 | I-BET-762 | GSK525762A | Chromatin other | BRD2, BRD3, BRD4 | 46943432 | | EVL,LRIG3,MACF1,PDIA5,SCYL3,SLC5A6,USP6NL, |  |
| 163 | JQ1 | JQ-1, (+)-JQ-1 | Chromatin other | BRD2, BRD3, BRD4, BRDT | 46907787 | | ARHGEF10,BACE2,CSMD3,DCC,FES,IL1B,PLP2,PRKACA,TCF3,XBP1,YME1L1, |  |
| 1218 | JQ1 | JQ-1, (+)-JQ-1 | Chromatin other | BRD2, BRD3, BRD4, BRDT | 46907787 | | EIF4EBP1,GABPB1,HMG20B,MALT1,POLB,SMARCE1,TXNRD1, |  |
| 1219 | PFI-1 | - | Chromatin other | BRD4 | 71271629 | | FKBP14,HOXC11,KDM5C,MYCBP,NISCH,NPDC1,RGS2,RRP1B,USP7, |  |
| 192 | LFM-A13 | DDE-28 | Other, kinases | BTK | 54676905 | | BCL3,BPHL,GRB7,IARS2,KAT6B,NR3C1,RPA1,TOP1,TP53BP2, |  |
| 331 | QL-X-138 | - | Other, kinases | BTK | none | | AURKA,B2M,BCL6,CSNK1E,CTNND1,PAK6,POLG2,PYCR1,RAB4A,SKI,TRAM2,WASF3, |  |
| 235 | QL-XII-47 | - | Other, kinases | BTK, BMX | none | | CYB561,EXT2,NUP98,PHGDH,PLOD3,PRKAR1A,PYGL,RFNG, |  |
| 1052 | RO-3306 | - | Cell cycle | CDK1 | 44450571 | | CRLF2,DECR1,H3F3A,MAP7,MYO5A,PCK2,RFWD3,SMARCE1,TCF7L2,WHSC1L1,ZNF384, |  |
| 219 | AT-7519 | AT7519 | Cell cycle | CDK1, CDK2, CDK4, CDK6, CDK9 | 11338033 | | BMP5,DRAP1,EML3,GAA,LRP10,NUMA1,PRAF2,TICAM1,TOR1A, |  |
| 53 | CGP-60474 | KIN001-019, CGP60474, CGP 60474 | Cell cycle | CDK1,CDK2,CDK5,CDK7,CDK9, PKC | 644215 | | HLA-A,IL2,KNSTRN,NSDHL,PLEKHJ1,RAB21,RPL39L,TBPL1,VAT1,WIF1, |  |
| 301 | PHA-793887 | PHA793887, PHA 793887 | Cell cycle | CDK2, CDK7, CDK5 | 46191454 | | BCL3,BCL7B,ITFG1,MDM2,ZNF586, |  |
| 110 | Seliciclib | Roscovitine, CYC-202, AL-39256 | Cell cycle | CDK2, CDK7, CDK9 | 160355 | | ACLY,AFF3,GLI2,IL7R,INPP1,SBDS,TBL1XR1,TRIM33,ZNF589, |  |
| 54 | CGP-082996 | CINK4, KIN001-021 | Cell cycle | CDK4 | 24825971 | | B4GAT1,CHD4,FAIM,NOLC1, |  |
| 1054 | Palbociclib | PD0332991, PD-0332991, PF-00080665-73 | Cell cycle | CDK4, CDK6 | 5330286 | | CCNC,ELN,JAK1,MYH11,NSDHL,NUP214,PRCC,RHOA,TAL1,TNFRSF21,VAPB, |  |
| 346 | THZ-2-102-1 | - | Cell cycle | CDK7 | none | | ATF5,CNBD1,CYLD,ELN,PTPRC,S100A7,TPR,TSPAN4, |  |
| 344 | THZ-2-49 | - | Cell cycle | CDK9 | none | | AXIN1,CCL2,EPHB2,IPO13,TNC,TOR1A, |  |
| 345 | KIN001-270 | CDK9 inhibitor, CDK9-IN-1 | Cell cycle | CDK9 | 66577006 | | CBLC,GLI2,PDS5A,PICALM,PPIE,TBC1D31,TIMM17B,UBE2L6, |  |
| 1022 | AZD7762 | AZD-7762, AZD 7762 | Cell cycle | CHEK1, CHEK2 | 11152667 | | APP,DCTD,FGFR2,KLHL21,PHKB,SMARCC1,TXLNA,VPS72,WHSC1L1, |  |
| 1020 | Lenalidomide | CDC-501, CC-5013, Revlimid | Protein stability and degradation | CRBN | 216326 | | COX6C,FCHO1,GLRX,LBR,NFKBIB,PLEKHJ1,RHEB,STAG1,STRN,VAV1, |  |
| 193 | GW-2580 | GX2580, CFMS receptor tyrosine kinase inhibitor | RTK signaling | CSF1R | 11617559 | | CDH3,CHST11,CNTRL,FAM135B,IER3,KLHDC2,LEF1,NTRK1,PDHX,TET1, |  |
| 199 | Pazopanib | Votrient | RTK signaling | CSF1R, KIT, PDGFRA, PDGFRB | 10113978 | | ATF1,CCR7,CHN1,CTNNA2,ELAVL1,ERBB3,ERCC3,MAPK9,SNX7,TSC1,ZNF384, |  |
| 329 | QL-XI-92 | - | Cytoskeleton | DDR1 | none | | CAMSAP2,CDC42,COG2,PCMT1,SCRN1,TLE1,TOMM34, |  |
| 71 | Pyrimethamine | Daraprim, Chloridine | Other | Dihydrofolate reductase (DHFR) | 4993 | | CHN1,EZH2,MUC16,NOL3,RPN1,SLC2A6,SLC37A4,SPDEF,TP53BP1,TXNDC9, |  |
| 1005 | Cisplatin | cis-Diammineplatinum(II) dichloride, Platinol, CIS-DDP | DNA replication | DNA crosslinker | 84691 | | BAD,BCORL1,CCDC6,CIITA,KDM6A,NBN,NUP214,SMC1A,SSBP2, |  |
| 136 | Mitomycin-C | Mytozytrex, NSC-26980, MMC, Mitosol, Mitozytrex | DNA replication | DNA crosslinker | 5746 | | CAST,EML3,KDM3A,PRCP,RARA,RHEB,SLC35B1, |  |
| 1038 | NU7441 | KU-57788, NU-7432, NU-7741 | Genome integrity | DNAPK | 11327430 | | COG7,HIST2H2BE,MEST,PER1,SFN,SMARCD1, |  |
| 1264 | SGC0946 | - | Chromatin histone methylation | DOT1L | 56962337 | | ARPP19,FANCG,HSPA4,MAP2K1,NCOA1,PDE4DIP,PIK3R3, |  |
| 1378 | Bleomycin (50 uM) | - | DNA replication | dsDNA break induction | 5460769 | | AXIN1,CLP1,CTCF,HMOX1,PLEKHJ1,SCRN1,TFG,ZNF274, |  |
| 190 | Bleomycin | - | DNA replication | dsDNA break induction | several | | ACD,AKT3,EZR,GADD45A,SBDS, |  |
| 1 | Erlotinib | Tarceva, RG-1415, CP-358774, OSI-774, Ro-508231, R-1415 | EGFR signaling | EGFR | 176870 | | CTSL,MSN,PACSIN3,RET,SLC5A6,TBX3,TPM4,TXNRD1, |  |
| 1114 | Cetuximab | Erbitux, IMC-C225, C225, IMC-225, L01XC06 | EGFR signaling | EGFR | 85668777 | | AKT2,BAZ1A,CISD1,DDB2,DHDDS,ECD,EIF4EBP1,ETS1,NIN,PCK2,PIGB,POT1,PTPN1,ROS1,SCCPDH, |  |
| 282 | Pelitinib | EKB-569, EKB 569 | EGFR signaling | EGFR | 6445562 | | CAMTA1,DCAF12L2,EIF5,MAPKAPK5,PEX11A, |  |
| 1010 | Gefitinib | ZD-1839, Iressa | EGFR signaling | EGFR | 123631 | | BAZ1A,BCL3,BCL7A,CHEK2,FEV,HACD3,MEF2C,PSMF1,RANBP2,RSPO2,SLC27A3,TPD52L2, |  |
| 1143 | HG-5-88-01 | - | Other, kinases | EGFR, ADCK4 | none | | CD320,CRKL,KIF5C,PRPF4,PTPN13,RFX5,SOCS2,TLK2, |  |
| 119 | Lapatinib | Tykerb, Tyverb | RTK signaling | EGFR, ERBB2 | 208908 | | BARD1,HOXA13,MUC16,PRAF2,TCF3,TLK2, |  |
| 1230 | IOX2 | IOX-2, IOX 2, AK176060 | Other | EGLN1 | 54685215 | | ARHGEF2,CDC20,ETV6,MET,NCOA2,RBM15,SMARCA4, |  |
| 111 | Salubrinal | EIF-2alpha Inhibitor | Other | EIF2A | 5717801 | | CLTC,MYH9,NPDC1,RABEP1, |  |
| 295 | NVP-BHG712 | BHG712 | RTK signaling | EPHB4 | 16747388 | | DFFA,DNAJB1,PER1,SCCPDH,UTP14A, |  |
| 255 | CP724714 | CP-724714 | RTK signaling | ERBB2 | 9874913 | | ARAF,ATP2B3,CTNND2,KDM3A,MRPS16,MSN,PXN,SLC35F2, |  |
| 1032 | Afatinib | BIBW2992, Tovok, Gilotrif | EGFR signaling | ERBB2, EGFR | 10184653 | | BIRC2,CDH1,GTF2A2,NBEA,PAFAH1B3,SYPL1, |  |
| 1377 | Afatinib | BIBW2992, Tovok, Gilotrif | EGFR signaling | ERBB2, EGFR | 10184653 | | BRIP1,CALU,CARS,CYLD,DMTF1,HK1,IL1B,IL2,ILK,NFATC4,PIK3CB,PRKCQ,SCARB1,ST7,TIMM17B,TMEM97, |  |
| 263 | FR-180204 | FR 180204, FR180204, ERK Inhibitor II | ERK MAPK signaling | ERK1, ERK2 | 11493598 | | ACAT2,CDC73,CHEK2,HSPD1,ID2,LPP,TESK1,VTI1A, |  |
| 158 | PF-562271 | PF-00562271 | Cytoskeleton | FAK, FAK2 | 11713159 | | CUL3,EGFR,ELAVL1,NCOR1,NR2F6,NT5C2,SYNGR3,TNIP1, |  |
| 166 | FTI-277 | - | Other | Farnesyl-transferase (FNTA) | 3005532 | | ARID1A,ASAH1,BDH1,CASP3,CDH3,CRTAP,INPP4B,POLE,PPIC,PTPN12,TNIP1,TXNL4B,WDR7, |  |
| 1049 | PD173074 | PD-173074, PD 173074 | RTK signaling | FGFR1, FGFR2, FGFR3 | 1401 | | ALDOA,ATP1A1,CDCA4,CSNK1A1,KIF14,TUBB6,WDR7, |  |
| 254 | Quizartinib | AC220, AC 220, AC-220, Asp-2689 | RTK signaling | FLT3 | 24889392 | | CBFB,ERBB4,FH,FLNA,GALE,IL1B,LAGE3,LAP3,NPC1,NUP214,PAFAH1B1,PHGDH,RNMT, |  |
| 1024 | Lestaurtinib | CEP-701, SP-924, SPM-924, A-154475, KT-555 | Other, kinases | FLT3, JAK2, NTRK1, NTRK2, NTRK3 | 126565 | | C5,KLK2,LIFR,LSM5,MYCL,ST3GAL5,SUPV3L1,TGFBR2,TP53BP2,TRAM2,TSPAN3, |  |
| 154 | CHIR-99021 | CT 99021, CHIR99021, CHIR 99021 | WNT signaling | GSK3A, GSK3B | 9956119 | | ARHGEF10,DDX3X,HMGN2P46,MACF1,MVP,ZRSR2 |  |
| 1241 | CHIR-99021 | CT 99021, CHIR99021, CHIR 99021 | WNT signaling | GSK3A, GSK3B | 9956119 | | AKAP8L,FH,FLNA,GRB10,JADE2,KIF5B,OXCT1,TBL1XR1,TCL1A,TMEM109, |  |
| 1025 | SB216763 | SB-216763, SB 216763 | WNT signaling | GSK3A, GSK3B | 176158 | | CAT,CCNE2,DDB2,DUSP6,GLI2,GLRX,KDSR,NUMA1,PDIA5,PLK1,PSMG1,SLC5A6,TFDP1, |  |
| 200 | Dacinostat | NVP-LAQ824, LAQ824 | Chromatin histone acetylation | HDAC1 | 6445533 | | ARHGEF10L,BAMBI,CCNF,DEK,EGF,ERC1,LSM14A,POLG2,RAB4A,RRAGA,SLC35A1,TPM1,ZDHHC6, |  |
| 272 | AR-42 | HDAC-42, AR 42, AR42 | Chromatin histone acetylation | HDAC1 | 6918848 | | FBXO7,FCGR2B,IDH1,MYL9,NPC1, |  |
| 89 | Parthenolide | - | Chromatin histone acetylation | HDAC1 | 7251185 | | COL2A1,CREG1,DFFB,FANCC,FOS,INTS3,MAP2K5,NIN,RHOH,RPL39L,TOMM70, |  |
| 274 | Belinostat | PXD101, PXD-101 | Chromatin histone acetylation | HDAC1 | 6918638 | | ACVR1,CEP89,CREG1,FANCC,NET1,PNP,PPP1R13B,WIF1,YME1L1, |  |
| 164 | JQ12 | - | Chromatin histone acetylation | HDAC1, HDAC2 | none | | DICER1,GNPDA1,KLF4,MAP4K4,MAPKAPK3,TERF2IP, |  |
| 88 | Entinostat | MS-275 | Chromatin histone acetylation | HDAC1, HDAC3 | 4261 | | ARID4B,CDC73,CEBPZ,MBNL2,PDGFRA,SMC1A,UBR5,WFS1, |  |
| 276 | CAY10603 | - | Chromatin histone acetylation | HDAC1, HDAC6 | 24951314 | | APP,AXIN2,CSF1R,ECT2L,JMJD6,LEF1,MTF2,NPDC1,RAB31,SMAD4,STIL,WASF3, |  |
| 273 | CUDC-101 | CUDC 101 | Other | HDAC1-10, EGFR, ERBB2 | 24756910 | | DROSHA,DUSP3,HTATSF1,LMO2,MKL1,STAP2,STAT1, |  |
| 165 | DMOG | Dimethyloxalylglcine | Metabolism | HIF-PH | 560326 | | B2M,BLM,CBFB,CHAC1,CORO1A,EIF1AX,FHL2,IFNAR1,KLHL21,PIM1,RAI14,SKI,TP63, |  |
| 1031 | Elesclomol | STA-4783 | Protein stability and degradation | HSP90 | 300471 | | BACE2,CTNND1,JUN,MLF1,PCMT1,RHOH,SFN, |  |
| 1170 | CCT-018159 | CCT018159, CCT 018159 | Protein stability and degradation | HSP90 | 5327091 | | CBR3,CREB1,PRCP,PRKCB,RABEP1,RTN2,TCTN1,WWTR1, |  |
| 328 | SNX-2112 | SNX 2112 | Protein stability and degradation | HSP90 | 24772860 | | ARHGEF10L,ATRX,BARD1,GADD45B,MSRA,MYCN,PALB2,SNX11,SS18,TET2,TIMM22,TP63, |  |
| 194 | Luminespib | AUY922, VER-52296,NVP-AUY922, AUY | Protein stability and degradation | HSP90 | 10096043 | | ATP1B1,ELL,ESR1,ORC1,PCMT1,PPIC,SORBS3,TIPARP, |  |
| 62 | BMS-536924 | BMS 536924 | IGF1R signaling | IGF1R, IR | 10390396 | | ACLY,BCL2,COPB2,CRK,GNAI1,KAT7,POLE,PSMD10,SBDS, |  |
| 184 | BMS-754807 | BMS754807, BMS 754807 | RTK signaling | IGF1R, IR | 24785538 | | ABL1,CFLAR,DNMT3A,EVL,NNT,NUP214,PALB2,RRP8,SACM1L, |  |
| 202 | GSK1904529A | GSK-1904529A, GSK 1904529A | RTK signaling | IGF1R, IR | 25124816 | | BCOR,CCNE2,DUSP3,DYRK3,KMT2C,MUC4,P2RY8,PHKG2,PLOD3,PRKAG2,SH3GL1,TP53, |  |
| 1091 | BMS-536924 | BMS 536924 | IGF1R signaling | IGF1R, IR | 10390396 | | CD44,COG2,FAS,PRKX,TCF3, |  |
| 91 | GSK319347A | KIN001-135, IKK-3 inhibitor | Other, kinases | IKK | 11626927 | | TFEB,TRIB1, |  |
| 203 | BMS-345541 | BMS345541, IKK Inhibitor 3 | Other, kinases | IKK1, IKK2 | 9813758 | | CASP3,KIAA0753,LMO1,NCKIPSD,SLC35B1,SMARCC1,TBPL1,TRIB1, |  |
| 305 | TPCA-1 | - | Other, kinases | IKK2 | 9903786 | | CERK,CPNE3,FOXL2,H3F3A,MYCL,PDHX,PRDM16, |  |
| 290 | KIN001-260 | Bayer IKKb inhibitor, ACHP | Other, kinases | IKKB | 10451420 | | AURKB,BCL3,BLM,CPNE3,EPN2,FANCF,GLOD4,KRAS,LMO2,NAB2,POLG2,SLC1A4,TET2,TXNDC9, |  |
| 1243 | Piperlongumine | Piplartine | Other | Induces reactive oxygen species | 637858 | | CRTC1,DNAJC15,EPB41L2,PPIE,RHOA,TIMM22,TLR4, |  |
| 63 | BMS-509744 | KIN001-127, ITK inhibitor | Other, kinases | ITK | 20635522 | | CTNND1,EPRS,EWSR1,FLI1,FOXO3,KDM6A,MFSD10,MLLT11,RRP8, |  |
| 206 | Ruxolitinib | INCB-18424, Ruxolitinib Phosphate, Jakafi | Other, kinases | JAK1, JAK2 | 25126798 | | AKAP8L,APOE,ASXL1,FOXO4,FZD1,KTN1,LMO2,NBEA,PRR7,TCTA, |  |
| 306 | Fedratinib | TG101348, TG-101348, SAR302503, SAR-302503 | Other, kinases | JAK2 | 16722836 | | ARHGAP1,ASPSCR1,BLMH,CDH17,CSNK1A1,CYP2C8,FAT3,ITGB1BP1,P4HA2,PMAIP1,PMS1,POP4,PTK2,TIMM17B,TLX1, |  |
| 1043 | JNK Inhibitor VIII | - | JNK and p38 signaling | JNK | 11624601 | | CCNA1,DMTF1,SMC1A, |  |
| 207 | AS601245 | - | JNK and p38 signaling | JNK1, JNK2, JNK2 | 10109823 | | A1CF,FZD1,MACF1,NF2,PTPRF,ZCCHC8, |  |
| 157 | JNK-9L | KIN001-204, JNK inhibitor 9l | JNK and p38 signaling | JNK2, JNK3 | 25222038 | | ABHD6,ACBD3,ASXL2,CCL2,CCND3,FOXP1,GATA2,KDM3A,SCRN1,VAV1, |  |
| 41 | S-Trityl-L-cysteine | NSC 83265, Tritylcysteine | Mitosis | KIF11 | 76044 | | APPBP2,BMPR1A,CIC,CIITA,FDFT1,FOSL1,FUBP1,PDHX,PWP1,SKI,TRRAP,WIPF2, |  |
| 298 | OSI-930 | OSI 930 OSI930 | RTK signaling | KIT | 9868037 | | CIAPIN1,CSRP1,HOXC13,MECOM,PAK6,PCNA,PNP,PWWP2A,PYGL,RMI2,USP44,VAV3, |  |
| 293 | Amuvatinib | MP470, MP 470, MP-470 | RTK signaling | KIT, PDGFRA, FLT3 | 11282283 | | BID,CD44,DCAF12L2,HLA-DRA,LIFR,MED12,PLAG1,RARA,RRP8,SHC1,STAT6,TRIB3,ZCCHC8, |  |
| 292 | Masitinib | AB1010, Masivet | RTK signaling | KIT, PDGFRA, PDGFRB | 10074640 | | CETN3,NNT,SLC27A3,TBXA2R, |  |
| 208 | Ispinesib Mesylate | SB-715992 | Mitosis | KSP | 6450816 | | CHCHD7,DDR1,LCK,NIPSNAP1,PLEKHJ1,SPDEF, |  |
| 256 | JW-7-24-1 | - | Other, kinases | LCK | none | | ABHD4,AKT1,TFRC,TOMM34,U2AF1, |  |
| 55 | A-770041 | KIN001-111 | Other, kinases | LCK, FYN | 9549184 | | ADRB2,DDR2,ELAVL1,FHL2,GLRX,HEY1,HSP90AA1,ISX,KIAA1549,PML,PTK2,SUPV3L1, |  |
| 1142 | HG-5-113-01 | - | Other | LOK, LTK, TRCB, ABL(T315I) | none | | MYLK,SIRT3,SRC,ZBTB16, |  |
| 333 | T0901317 | TO-901317, TO901317 | Other | LXR, FXR | 447912 | | CARS,CCR4,DUSP4,EGR1,EML4,FBXL12,PAK4,PDGFRA,PROS1,RALA,SGK1,SOX4,SYNE2,TIMELESS, |  |
| 291 | KIN001-266 | - | ERK MAPK signaling | MAP3K8 | 44143370 | | ATMIN,CIITA,EAPP,MDM2,MEF2C,PALB2,USP44, |  |
| 1133 | Serdemetan | JNJ-26854165 | p53 pathway | MDM2 | 11609586 | | ATP6V0B,CCDC92,CHST11,DLD,ITFG1,KIF14,PARP1,ZNF131, |  |
| 1047 | Nutlin-3a (-) | - | p53 pathway | MDM2 | 11433190 | | CSNK1A1,DERA,ITGB1BP1,LRP10,MALT1,MYLK,NSDHL, |  |
| 269 | NSC-207895 | XI-006, NSC207895 | p53 pathway | MDM4 | 42640 | | CALU,CANT1,CHN1,CHP1,EBP,GLRX,MYO5A,NAB2,NCKIPSD,NFKBIA,PLP2,PTPN12,TIMM17B, |  |
| 1062 | Selumetinib | AZD6244, AZD-6244, ARRY-886 | ERK MAPK signaling | MEK1, MEK2 | 10127622 | | DDX6,EGFR,KIF2C,NRAS,SKI,TBP,TBPL1,TP53,WAS, |  |
| 1498 | Selumetinib | AZD6244, AZD-6244, ARRY-886 | ERK MAPK signaling | MEK1, MEK2 | 10127622 | | ALDH7A1,FOXA1,GRWD1,KCNK1,LSM14A,PACSIN3,PGRMC1,PSMF1,TICAM1, |  |
| 1060 | PD0325901 | PD-0325901, PD 0325901 | ERK MAPK signaling | MEK1, MEK2 | 9826528 | | ATP2B3,BCLAF1,BDH1,CNDP2,HMGCR,IER3,KIF20A,MEF2C,NCOA1,NOSIP,SCYL3,TCFL5,VAV1,ZNF589, |  |
| 1014 | Refametinib | RDEA119, BAY-86-9766, BAY 869766 | ERK MAPK signaling | MEK1, MEK2 | 44182295 | | CD40,EDN1,ERBB3,FEZ2,HOOK3,HOXD13,KLHL21,NAB2,NCAPD2,NFKBIB,PRR15L,SCAND1,XBP1,ZDHHC6, |  |
| 1015 | CI-1040 | CI 1040, PD-18435, PD-184352, 212631-79-3 | ERK MAPK signaling | MEK1, MEK2 | 6918454 | | CHD4,EDN1,ENOSF1,IGF1R,MTHFD2,PLK1, |  |
| 1526 | Refametinib | RDEA119, BAY-86-9766, BAY 869766 | ERK MAPK signaling | MEK1, MEK2 | 44182295 | | DNM1L,MOK,NTHL1,PPIC,SLC25A4,STAMBP,VAPB, |  |
| 279 | BIX02189 | BIX 02189 | ERK MAPK signaling | MEK5, ERK5 | 46931012 | | CRTC1,FGFR3,HLA-DRA,MLLT4,SPRED2, |  |
| 6 | PHA-665752 | PHA665752, PHA 665752 | RTK signaling | MET | 10461815 | | ACBD3,ARHGAP1,ARID1A,CBLB,CD40,FBXO21,GATA1,IL21R,NCOR2,PDHX,PTPRK,SOX21,TCL1A,WDR61, |  |
| 37 | Crizotinib | Xalkori, PF2341066, PF-2341066, PF 2341066 | RTK signaling | MET, ALK, ROS1 | 11626560 | | BNIP3,CCDC86,GATA2,MCOLN1,NCKIPSD,NIN,NUP214,P4HA2,SIX1,ZNF451, |  |
| 308 | Foretinib | GSK1363089, XL-880, EXEL-2880, GSK089 | RTK signaling | MET, KDR, TIE2, VEGFR3/FLT4, RON, PDGFR, FGFR1, EGFR | 42642645 | | ABHD4,ANK1,CREBBP,ELN,FOXL2,IGHMBP2,PDHX,RAB27A,SLC35A3,SLC35B1, |  |
| 1007 | Docetaxel | RP-56976, Taxotere | Mitosis | Microtubule stabiliser | 148124 | | B4GAT1,DUSP3,FAS,HADH,ISX,KAT6B,KDM3A,PLEKHJ1,RANBP2,RFWD3,SOX4,TRIM27, |  |
| 11 | Paclitaxel | BMS-181339-01, Taxol, Onxol, Paxene, Praxel, Abraxane | Mitosis | Microtubule stabiliser | 36314 | | CD209,ETV5,HMOX1,JAZF1,PRKCD,RSU1,SLC2A6,STAT3,TCTA, |  |
| 201 | Epothilone B | Patupilone, EpoB, EPO906, GNF-PF-193 | Mitosis | Microtubule stabiliser | 448013 | | ARPP19,CALU,HSPA4,NBEA,PNP,RAC1,RFWD3,TBC1D9B,TNC,USP6NL,VTI1A,ZMIZ1, |  |
| 294 | MPS-1-IN-1 | - | Mitosis | MPS1 | 25195352 | | ANXA7,ARID4B,CNBD1,CSRP1,ECH1,FPGS,PLEKHJ1,SLC35F2, |  |
| 83 | JW-7-52-1 | NA | PI3K/MTOR signaling | MTOR | 49836027 | | B4GAT1,BIRC2,FZD7,HEATR1,PTPRK,RFWD3,SACM1L,SUZ12,TCF7L2,UGDH, |  |
| 1166 | QL-VIII-58 | - | Other | MTOR, ATR | none | | CDK1,CSNK1E,ERCC3,ERG,FOXJ3,NBN,SOCS2,TFDP1, |  |
| 3 | Rapamycin | AY-22989, Sirolimus, WY-090217, Torisel, Rapamune | PI3K/MTOR signaling | MTORC1 | 5384616 | | BID,C2CD5,CCDC86,RNMT, |  |
| 299 | OSI-027 | A-1065-5 | PI3K/MTOR signaling | MTORC1, MTORC2 | 44224160 | | BRAF,CDC45,CTNND2,EPB41L2,FAT1,GAS7,HSPA4,MYCBP2,PAK6,SLC11A2, |  |
| 1059 | AZD8055 | AZD-8055 | PI3K/MTOR signaling | MTORC1, MTORC2 | 25262965 | | B4GAT1,CARS,ERBB3,NCOA3,NIN,POT1, |  |
| 1529 | Pevonedistat | MLN4924, MLN 4924, MLN-4924 | Other | NAE | 16720766 | | ATM,CHP1,MYO10,RAF1,SKP1, |  |
| 1248 | Daporinad | APO866, FK866, FK866 | Metabolism | NAMPT | 6914657 | | FANCG,FOXO3,HMGA2,MDM2,MSH6,NTRK1,RMI2,SMARCD1, |  |
| 1266 | ICL1100013 | - | Other | N-myristoyltransferase 1/2 | - | | ATP6V1D,BAMBI,CLTCL1,FAM135B,FNBP1,IGF2R,MAMLD1,MTFR1,NUP88,NUTM1,VAPB,ZNF131, |  |
| 211 | TL-2-105 | - | Other | not defined | none | | CALR,FAT1,HLA-DRA,LARP4B,MN1,MTHFD2,NUP133,RAD51B,RFX5,VAV1, |  |
| 170 | Shikonin | Anchusin | Other | not defined | 5208 | | FAM131B,FAM20B,GATA3,ISX,MELK,PCBP1,PDGFA,SLC2A6, |  |
| 1023 | GW441756 | GW 441756 | RTK signaling | NTRK1 | 9943465 | | CNBD1,H2AFV,NTRK1,PCNA,PTPN13,TFAP2A, |  |
| 1042 | Doramapimod | BIRB-796, BIRB 796 | JNK and p38 signaling | p38, JNK2 | 156422 | | FOXL2,HES1,IGF2R,TLX3, |  |
| 221 | TAK-715 | KIN001-201, TAK 715 | JNK and p38 signaling | p38alpha, p38beta | 9952773 | | ARID4B,CDC25B,NCOA2,PXN,RFC5,SETD2,SH2B3,STX4,TBX2, |  |
| 176 | IPA-3 | IPA 3 | Cytoskeleton | PAK1 | 521106 | | ARHGEF10L,HLF,HSPA4,IARS2,MLLT1,NRAS,TOMM70,TSC1,XPC, |  |
| 1175 | Rucaparib | PF-01367338, AG-014699, AG-14447, AG-14699 | Genome integrity | PARP1, PARP2 | 9931953 | | ARID4B,CENPE,DCAF12L2,EXT1,FANCC,RFWD3,SBDS,SS18,TGFBR2,TM9SF2,WNK2, |  |
| 1259 | Talazoparib | BMN-673, BMN 973 | Genome integrity | PARP1, PARP2 | 44819241 | | AGL,CCNE2,DAG1,DDX6,HS2ST1,LYRM1,PRKCB,PRPF4,TSPAN4, |  |
| 1017 | Olaparib | AZD2281, KU0059436, Lynparza | Genome integrity | PARP1, PARP2 | 23725625 | | C2CD5,C5,EIF3E,GRWD1,IL1B,IL6ST,MAPK1,RAP1GDS1,SIX2,TP53, |  |
| 1495 | Olaparib | AZD2281, KU0059436, Lynparza | Genome integrity | PARP1, PARP2 | 23725625 | | CPSF4,FBXW7,GLRX,HOMER2,ITGB1BP1,LMO1,PAK4,PPFIBP1,TP53BP1, |  |
| 5 | Sunitinib | Sutent, Sunitinib Malate, SU-11248 | RTK signaling | PDGFR, KIT, VEGFR, FLT3, RET, CSF1R | 5329102 | | FLT4,FOXA1,LRIG3,MAMLD1,MIF,MLLT11,PDGFA,RFNG,RNMT,SGK1,SLC11A2,STRN, |  |
| 30 | Sorafenib | Nexavar, 284461-73-0, BAY 43-9006 | RTK signaling | PDGFR, KIT, VEGFR, RAF | 216239 | | ACVR1,LARP4B,PRDM2,SCYL3,TMPRSS2, |  |
| 167 | OSU-03012 | AR-12, OSU 03012, OSU03012, PDK1 inhibitor AR-12 | Metabolism | PDK1 (PDPK1) | 10027278 | | CASC5,COL2A1,DUSP11,FGFR4,HEY1,NET1,NUP133,OMD,PMAIP1,PTPRF,RABEP1,TUBB6, |  |
| 287 | KIN001-244 | PDK1 inhibitor 7 | Metabolism | PDK1 (PDPK1) | 56965967 | | CANT1,CDC45,EP300,FLT3,HOXD13,PHF6,RAC1,RPN1,ZNF451, |  |
| 222 | BX-912 | - | Metabolism | PDK1 (PDPK1) | 11754511 | | CASP7,CIRBP,CNPY3,LMNA,NCK2,PRRX1,PTK2B,RSU1,TRAPPC3,TXNRD1, |  |
| 1058 | Pictilisib | GDC-0941, GDC0941, RG-7621 | PI3K/MTOR signaling | PI3K (class 1) | 17755052 | | CCNB1IP1,CXCL2,DLD,DNM2,GNA11,MUC16,PPP2R3C,QKI,SLC35F2,TBP,XBP1,ZNF429, |  |
| 1527 | Pictilisib | GDC-0941, GDC0941, RG-7621 | PI3K/MTOR signaling | PI3K (class 1) | 17755052 | | CCNA1,CNOT4,GLI2,ICAM1,IKZF1,KIAA0753,MAP2K1,PHKA1,POT1,SUV39H1, |  |
| 1057 | Dactolisib | NVP-BEZ235, BEZ235 | PI3K/MTOR signaling | PI3K (class 1), MTORC1, MTORC2 | 11977753 | | ACKR3,AKT3,ARPP19,CD79A,MECOM,PHGDH,PTPN6,RAB4A,SND1,TFEB,TSC22D3, |  |
| 283 | Omipalisib | GSK2126458, GSK-2126458, EX-8678, GSK458 | PI3K/MTOR signaling | PI3K (class 1), MTORC1, MTORC2 | 25167777 | | BZW2,HMOX1,SETBP1,SMARCC1, |  |
| 302 | PI-103 | PI-103, PI103, PI 103 | Other, kinases | PI3Kalpha, DAPK3, CLK4, PIM3, HIPK2 | 9884685 | | DROSHA,PARP2,PTK2B,SMARCC1,THAP11,TLK2, |  |
| 94 | TGX221 | TGX-221, Tgx 221 | PI3K/MTOR signaling | PI3Kbeta | 9907093 | | BZW2,CCNF,FZD7,MSN,NUP93,TMEM109,TOMM34, |  |
| 1066 | AZD6482 | AZD 6482, AZD-6482, AK-55409 | PI3K/MTOR signaling | PI3Kbeta | 44137675 | | ABI1,FEZ2,POT1,SMARCE1, |  |
| 156 | AZD6482 | AZD 6482, AZD-6482, AK-55409 | PI3K/MTOR signaling | PI3Kbeta | 44137675 | | CHST11,FZD7,GNA11,NCK2,PSMG1,SPR,STAT5B,TFDP1, |  |
| 238 | Idelalisib | CAL-101, Zydelig | PI3K/MTOR signaling | PI3Kdelta | 11625818 | | CTNNAL1,KRAS,ME2,MUTYH,NFATC3,PPP2R1A,TCFL5,TIAM1, |  |
| 224 | AS605240 | KIN001-173, AS-605240 | PI3K/MTOR signaling | PI3Kgamma | 5289247 | | ARHGAP26,ARHGAP5,CSF3R,ELF4,GPC5,GRB7,IDH2,IQGAP1,KMT2D,MAN2B1,NDRG1,RAD51B,SLC5A6,SSX1,TFG, |  |
| 303 | PIK-93 | PIK 93, PIK93 | PI3K/MTOR signaling | PI3Kgamma | 6852167 | | ARHGEF10,BDH1,LASP1,NR3C1,RAP1GAP,SNX11, |  |
| 197 | Bryostatin 1 | Bryostatin | Other | PKC | 5280757 | | CASC3,POLD1, |  |
| 153 | Midostaurin | PKC412, benzoylstaurosporine, CGP-41251 | Other | PKC, PPK, FLT1, c-FGR, others | several | | AMER1,ARID4B,CDH11,FBXL12,FOXJ3,HK1,HMGA1,MRPL19,NONO,PRDM1,USP8, |  |
| 229 | Enzastaurin | LY317615 | Other, kinases | PKCB | 176167 | | CPEB3,DPH2,ECD,FSD1,GAA,HOXA11,HYOU1,IL2,KLHL21,LMO1,PAF1,ROBO2,TET1,TRIM24,TRIP11, |  |
| 87 | GW843682X | GW843682X (AN-13) | Cell cycle | PLK1 | 9826308 | | CORO1A,DDX3X,ICMT,LBR,LPP,MSH6,NIPSNAP1,PAK1,PRKAG2,PRKX,RTN2,SH2B3,TBC1D9B,TCF7L2,TIMELESS, |  |
| 60 | BI-2536 | - | Cell cycle | PLK1, PLK2, PLK3 | 11364421 | | AKT1,GRWD1,HLA-A,INPP4B,LYN,MBTPS1,NUP133,PDHX,PTPN1,TJP1, |  |
| 257 | NPK76-II-72-1 | - | Cell cycle | PLK3 | none | | AFF4,ELAC2,HOOK3,MUC4,TERF2IP, |  |
| 173 | FH535 | - | Other | PPARgamma, PPARdelta | 3463933 | | CETN3,ELF4,ERCC4,FCRL4,FGFR1,GNB5,ME2,MYB,NCKIPSD,PRRX1, |  |
| 1067 | CCT007093 | - | Cell cycle | PPM1D | 2314623 | | LIFR,MUC1,NUP88,PTPN1,PTPRF,WWTR1, |  |
| 175 | PAC-1 | GTPL5238 | Apoptosis regulation | Procaspase-3, Procaspase-7 | 6753378 | | ANK1,ASXL2,ATR,AURKA,BCL7A,DUSP4,LRRC41,MYC,SLC11A2,TRAK2,ZRSR2 |  |
| 104 | Bortezomib | PS-341, LDP-341, Velcade | Protein stability and degradation | Proteasome | 387447 | | COX6C,FPGS,H2AFV,ITGB1BP1,LAP3,MLLT10,NUP133,PNKP,SLC37A4,SQSTM1,USP14,XPC, |  |
| 9 | MG-132 | LLL cpd, MG 132, MG132 | Protein stability and degradation | Proteasome, CAPN1 | 462382 | | DNAJB2,ETNK1,RALB,TLX3,ZNF429, |  |
| 135 | Gemcitabine | Gemzar, LY-188011 | DNA replication | Pyrimidine antimetabolite | 60750 | | CDC42,CDH11,CHEK2,CSNK1E,ELN,GLI1,GPER1,LBR,LHFP,LYRM1,PLK1,PTK2B,RGS2,SOX4,TES,TRIB3, |  |
| 1069 | EHT-1864 | EHT 1864 | Cytoskeleton | RAC1, RAC2, RAC3 | 9938202 | | BCL3,ERCC4,LSM14A,MBOAT7,MDM2,NUDT9,PTCH1,RGS7,SLC35B1,TCF3,ZMIZ1, |  |
| 186 | Bexarotene | LG-100069, Targretin, Targret, Targrexin, Targretyn, Bexarotenum | Other | Retinioic X receptor (RXR) agonist | 82146 | | EZH2,IL6ST,ITGB5,MYCBP2,SCRN1,SDHA,SFPQ, |  |
| 300 | CX-5461 | CX5461, CX 5461 | Other | RNA Polymerase 1 | 25257557 | | ANO10,EPAS1,ETFB,EXT2,HMGA2,MALAT1,MBTPS1,NIT1,RNF167,TBPL1,TCEA1,TMEM109,VTI1A, |  |
| 127 | GSK269962A | GSK 269962A | Cytoskeleton | ROCK1, ROCK2 | 16095342 | | ASAH1,CNTRL,HLA-DRA,IRS4,ITGAV,JUN,LBR,PYCR1,TCTN1,ZNF384, |  |
| 1192 | GSK269962A | GSK 269962A | Cytoskeleton | ROCK1, ROCK2 | 16095342 | | ACD,ARID5B,DPH2,DYNLT3,NCOA2,NISCH,NRG1,SFPQ,USP1,ZNRF3, |  |
| 230 | GSK429286A | - | Cytoskeleton | ROCK1, ROCK2 | 11373846 | | ARHGAP5,ASXL1,BCL9L,CASP9,CBLC,CCR4,CYTH1,DUSP4,GAS7,HMGA2,KIAA0753,NUDCD3,PECR,PNKP,PNP,SCAND1, |  |
| 231 | FMK | KIN001-242 | Other, kinases | RSK | none | | ABCF1,CAST,CGRRF1,NOS3,SMNDC1,ZNF589, |  |
| 1039 | SL0101 | SL-0101, SL 0101-1 | Other, kinases | RSK, AURKB, PIM1, PIM3 | 10459196 | | CAB39,CACNA1D,DNTTIP2,DYNLT3,GTF2A2,HEATR1,MCM3,PBX1,SKP1, |  |
| 64 | CMK | KIN001-128 | Other, kinases | RSK2 | 16663089 | | CRTC3,CSMD3,FBXW7,GPHN,KCNK1,PAX3,SIRT3,TIMM17B,TSC1,WDTC1, |  |
| 1129 | PF-4708671 | PF 4708671, PF4708671 | PI3K/MTOR signaling | S6K1 | 51371303 | | BCL11B,GFOD1,KAT6B,KLHL21,MYH11,PLSCR1,RPL22,TFE3,TMCO1, |  |
| 177 | GSK650394 | GSK-650394, GSK 650394 | Other, kinases | SGK2, SGK3 | 25022668 | | CD274,FEV,MAPKAPK3,PIK3C3,PIN1,PRDM1,TXNL4B,USP44, |  |
| 147 | NSC-87877 | NSC 87877 | Other | SHP-1 (PTPN6), SHP-2 (PTPN11) | 5459322 | | ARHGAP26,PPFIBP1,QKI,STAT6,VAPB, |  |
| 341 | Selisistat | EX-527, EX 527 | Chromatin histone acetylation | SIRT1 | 5113032 | | ATRX,BID,CDCA4,DYNLT3,FANCE,FAT4,IDH1,MAN2B1,PNKP,SUPV3L1,USP44, |  |
| 1530 | PFI-3 | BDF00016096 | Chromatin other | SMARCA2, SMARCA4, PB1 | 78243717 | | ABL1,CDCA4,GDPD5,KIAA1549,MLLT3,MSI2,NUDCD3,PALB2,RPS5,S100A7,SH3GL1,SOCS1,STXBP1,TERF2IP,XPO1, |  |
| 17 | Cyclopamine | - | Other | SMO | several | | ACBD3,PSMF1,SMC3,SSX1,TBL1XR1,THRAP3, |  |
| 1019 | Bosutinib | SKI-606, Bosulif | Other, kinases | SRC, ABL, TEC | 5328940 | | CTNNAL1,E2F2,GATA1,GLOD4,H2AFV,LOXL1,STAP2,TCERG1, |  |
| 178 | BAY-61-3606 | Syk Inhibitor, BAY-613606 | Other, kinases | SYK | 10200390 | | ADH5,C2CD2,CD28,ERO1A,FANCC,HAT1,PLK1,RAI14,TP53BP2, |  |
| 261 | TL-1-85 | - | Other, kinases | TAK | none | | CLIP1,CRYZ,FAM131B,FDFT1,IKZF1,KNSTRN,MAP2K1,MBTPS1,SDHB,STAG2, |  |
| 1242 | (5Z)-7-Oxozeaenol | 5Z-7-Oxozeaenol, LL-Z1640-2 | Other, kinases | TAK1 | 9863776 | | CIAPIN1,GADD45B,GALE,GAS7,GLI1,MAPK1IP1L,NBN,PIK3C3,PYCR1,SND1,TM9SF3, |  |
| 260 | NG-25 | NG25 | Other, kinases | TAK1, MAP4K2 | 53340664 | | EPHA3,MBNL2,OLIG2,RPN1,TEX10, |  |
| 1037 | BX795 | BX-795 | Other, kinases | TBK1, PDK1 (PDPK1), IKK, AURKB, AURKC | 10077147 | | CLP1,ERG,PDGFRB,PHKG2,PMS2,RHEB,STX4,VAT1,WT1, |  |
| 1194 | SB505124 | SB 505124, SB505124 | RTK signaling | TGFBR1, ACVR1B, ACVR1C | 9858940 | | BNIP3,CACNA1D,ERBB4,FGFR3,MLEC,PTEN,SOCS2, |  |
| 1494 | SN-38 | 7-Ethyl-10-Hydroxy-Camptothecin, SN 38 | DNA replication | TOP1 | 104842 | | BLCAP,CCNB1,CRTC1,DUSP4,FZD7,PROS1,RECQL4,VPS28, |  |
| 134 | Etoposide | Etopophos, Vepesid, Eposin, VP-16 | DNA replication | TOP2 | 36462 | | BID,CFLAR,CNDP2,IQGAP1,PER1,PROS1,RFC2,TPR, |  |
| 249 | Cabozantinib | BMS-907351, XL-184, Cometriq | RTK signaling | VEGFR, MET, RET, KIT, FLT1, FLT3, FLT4, TIE2,AXL | 25102847 | | ACAT2,ACLY,BCR,BLCAP,DDX6,EIF3E,FIS1,HACD3,KAT6A,PTPRD,TXLNA,UBR5, |  |
| 1029 | Motesanib | AMG-706, AMG 706, AMG706 | RTK signaling | VEGFR, RET, KIT, PDGFR | 11667893 | | CBR3,EP300,FES,IGF1R,JMJD6,LGALS8,PML,PYCR1,TFE3,USP6, |  |
| 277 | Linifanib | ABT-869, ABT 869 | RTK signaling | VEGFR1, VEGFR2, VEGFR3, CSF1R, FLT3, KIT | 11485656 | | CDKN2A,CETN3,CHCHD7,GPC5,HRAS,NUMA1,P4HTM,RAB31, |  |
| 172 | Embelin | Emberine, Embelic acid | Apoptosis regulation | XIAP | 3218 | | DNMT3A,IKBKB,IL21R,MBOAT7,POLR2I,SCRN1, |  |
